# A comprehensive analysis of SNPs and CNVs identifies novel markers associated with disease outcomes in colorectal cancer

**DOI:** 10.1002/1878-0261.13067

**Published:** 2021-08-05

**Authors:** Yajun Yu, Salem Werdyani, Megan Carey, Patrick Parfrey, Yildiz E. Yilmaz, Sevtap Savas

**Affiliations:** ^1^ Discipline of Genetics Faculty of Medicine Memorial University St. John’s NL Canada; ^2^ Discipline of Medicine Faculty of Medicine Memorial University St. John’s NL Canada; ^3^ Department of Mathematics and Statistics Faculty of Science Memorial University St. John’s NL Canada; ^4^ Discipline of Oncology Faculty of Medicine Memorial University St. John’s NL Canada; ^5^ Present address: Division of Biomedical Sciences Faculty of Medicine Memorial University St. John’s NL Canada

**Keywords:** colorectal cancer, genetic variants, genome‐wide association study, prognostic markers, proportional hazards (PH) assumption, variables with time‐varying associations

## Abstract

We aimed to examine the associations of a genome‐wide set of single nucleotide polymorphisms (SNPs) and 254 copy number variations (CNVs) and/or insertion/deletions (INDELs) with clinical outcomes in colorectal cancer patients (*n* = 505). We also aimed to investigate whether their associations changed (e.g., appeared, diminished) over time. Multivariable Cox proportional hazards and piece‐wise Cox regression models were used to examine the associations. The Cancer Genome Atlas (TCGA) datasets were used for replication purposes and to examine the gene expression differences between tumor and nontumor tissue samples. A common SNP (*WBP11‐*rs7314075) was associated with disease‐specific survival with *P*‐value of 3.2 × 10^−8^. Association of this region with disease‐specific survival was also detected in the TCGA patient cohort. Two expression quantitative trait loci (eQTLs) were identified in this locus that were implicated in the regulation of *ERP27* expression. Interestingly, expression levels of *ERP27* and *WBP11* were significantly different between colorectal tumors and nontumor tissues. Three SNPs predicted the risk of recurrent disease only after 5 years postdiagnosis. Overall, our study identified novel variants, one of which also showed an association in the TCGA dataset, but no CNVs/INDELs, that associated with outcomes in colorectal cancer. Three SNPs were candidate predictors of long‐term recurrence/metastasis risk.

## Graphical abstract

Stratifying patients with colorectal cancer (CRC) into high and low outcome risk groups is important for informing treatment and follow‐up strategies. Here, we examined the associations of a genome‐wide set of single nucleotide polymorphisms (SNPs) and a set of copy number variations and indels with clinical outcomes in patients with CRC. We showed that CRC patients can be distinguished for their risk of death from this disease based on their genotypes of a SNP in the *WBP11* gene.

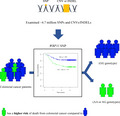

**Abbreviations**
BAF: B allele frequency
CI: confidence interval
CMS: consensus molecular subtypes
CN: copy number
CNV: copy number variation
DGV: Database of Genomic Variants
DSS: disease-specific survival
eQTL: expression quantitative trait locus
GDC: Genomic Data Commons
GWAS: genome-wide association study
HMM: hidden Markov model
HR: hazard ratio
HWE: Hardy–Weinberg equilibrium
IBS: identity-by-state
INDEL: insertion/deletion
LD: linkage disequilibrium
LRR: log R ratio
MAF: minor allele frequency
MDS: multidimensional scaling
MSI: microsatellite instability
MSI-H: microsatellite instability-high
MSI-L: microsatellite instability-low
MSS: microsatellite stable
NFCCR: Newfoundland Familial Colorectal Cancer Registry
NLCHI: Newfoundland and Labrador Center for Health Information
OR: olfactory receptor
PCA: principal component analysis
PFB: population frequency of B allele
PH: proportional hazard
QC: quality control
RMFS: recurrence/metastasis-free survival
SNP: single nucleotide polymorphism
TCGA: The Cancer Genome Atlas

## 1 Introduction

A significant portion of colorectal cancer patients die of this disease and develop local recurrences and metastases over time [1, 2]. Knowledge on the baseline predictors of clinical outcomes is essential for effective disease management. The disease stage is the most well‐known prognostic marker in colorectal cancer [3, 4]. Other factors, including tumor location, microsatellite instability status, and treatment, have also been associated with patient outcomes [5, 6, 7]. However, patients who are categorized in the same prognostic group may experience different outcomes, indicating the need for additional prognostic markers to distinguish between patients with different outcome risk. Given that genetics plays a role in many human phenotypes, it is intuitive to hypothesize that genetic variants can be prognostic markers in colorectal cancer.

A number of studies have examined the associations of genetic variations, such as SNPs, with clinical outcomes in colorectal cancer. While these studies focused mostly on candidate variant, gene, or pathway analyses [8, 9, 10, 11, 12, 13, 14, 15, 16, 17, 18, 19, 20], a small number of genome‐wide association studies (GWASs) were also performed [21, 22, 23, 24, 25, 26]. These GWASs focused on often diverse outcome measures, identified a limited set of variants and potential genes, and their results largely remain to be confirmed by further studies. SNPs are the most common genetic variables; however, human genome also contains copy number variants (CNVs; ≥ 1 kb) and insertion/deletion variants (INDELs; < 1 kb). While analysis of copy number alterations in tumor genomes is widely performed, there are not many studies that have checked the potential associations of germline CNVs/INDELs with survival outcomes in colorectal cancer [27, 28, 29, 30]. As a result, similar to SNP studies, only a handful genes and CNVs/INDELs have been identified as candidate prognostic markers in colorectal cancer.

Survival studies can identify prognostic markers that can predict the hazard over the follow‐up periods [31, 32, 33]. Normally, such markers can distinguish between patients with different outcome risk regardless of time. In rare cases, however, it has been shown that some markers have different levels or types of associations during different time‐periods of the follow‐up (i.e., time‐varying associations). Such markers, therefore, can help distinguish between patients with high and low outcome risk during certain time‐periods. For example, in our previous colorectal cancer study, prognostic associations became stronger, weaker, appeared, or diminished over time for a set of baseline clinical variables [34]. Similarly, we and others identified two somatic alterations [34, 35] and three genetic polymorphisms [24, 29] that were associated with early or late risk of disease outcomes in colorectal cancer. Knowledge on such markers is surprisingly limited. This may be because that many cohorts do not have long follow‐up times that are essential for identifying whether a variable has constant or time‐varying associations with outcomes.

This literature information indicates that further studies on genome‐wide sets of SNPs, CNVs/INDELs, and colorectal cancer outcomes are necessary to improve the current level of knowledge. In addition, there is a need for studies that investigate time‐varying associations, as this type of analysis provides unique insight into prognosis. In this study, we examined large sets of common genetic variants (˜ 4.7 million SNPs and 254 CNVs/INDELs) and their associations with disease‐specific survival and recurrence/metastasis‐free survival in a colorectal cancer patient cohort (*n* = 505 and 495, respectively) followed up to 19 years. Our objectives were to (a) investigate the associations of genetic variants with the outcomes, (b) examine whether any of the variants had time‐varying associations, and (c) further explore our findings using The Cancer Genome Atlas (TCGA) datasets for replication purposes and gene expression analyses.

## 2 Methods

### 2.1 Ethics approval

This study complied with the Declaration of Helsinki and was approved by the Human Research Ethics Board (HREB) of Newfoundland and Labrador (reference numbers: 2009.106; 2015.294; 2016.252). As this is a research study with a secondary use of data, HREB waived the consent requirement.

### 2.2 Patient cohort and clinical and genetic data

Patients in the Newfoundland Colorectal Cancer Registry (NFCCR) cohort were diagnosed between 1999 and 2003 and followed up to 19 years [34, 36, 37, 38]. The NFCCR patient cohort has been described in other publications [34, 36, 37]. A total of 750 patients were collected over 5 years (1999–2003). The last follow‐up date was January 2018 [34]. Clinical data were obtained from several resources, including medical charts, electronic medical records, Provincial Tumor Registry‐NL/Dr. H. Bliss Murphy Cancer Centre, and Newfoundland and Labrador Center for Health Information (NLCHI) [34, 37, 38]. Microsatellite instability (MSI) status was previously identified using tumor DNAs as explained in Woods *et al*. [37]. DNA samples extracted from white blood cells were available for 539 patients at the time of genotyping. Out of 539, patients who passed the sample quality control measures, satisfied the inclusion criteria [21], and had the genetic data available (SNP or CNV/INDEL genotype data) were included in the analyses. All patients included were Caucasians and unrelated to each other [21].

Genetic data examined in this study include two datasets [21, 29]. The SNP dataset, which is available for 505 patients (Table 1), includes 4 711 309 SNPs that qualified for analysis (genotyped SNPs = 607 365; imputed SNPs = 4 103 944). Genetic imputation was done using shapeit (v2.r837) [39] and impute2 (v2.3.2) [40], using the 1000 Genomes Phase 3 data [41] as the reference panel data. The initial SNP genotype data, inclusion/exclusion and quality control (QC) metrics, and imputation procedures are explained in detail as follows: The initial SNP genotype data were obtained using the Illumina^®^ Omni1‐Quad human SNP genotyping platform at an outsourced commercial facility (Centrillion Biosciences, USA) [21]. Data included 811 162 SNPs that met the following criteria: (a) SNPs that were successfully genotyped and with a missing rate ≤ 5%; (b) SNPs that satisfied the Hardy–Weinberg equilibrium (HWE; *P*‐value > 1 × 10^−04^); (c) SNPs with minor allele counts > 2; (d) in cases when multiple SNPs shared the same genomic position, SNPs with the rs numbers were retained; and (e) SNPs that were on the autosomal chromosomes. plink v1.07 [42] was used to extract these data from the original datafiles. These SNP data were then used in a genetic imputation process using the software shapeit (v2.r837) [39] and impute2 (v2.3.2) [40] (for details, see SNP imputation; Figs S1–S3). Quality control measures were applied to variants: info scores of imputed SNPs > 0.7, maximum probability of the imputed genotypes > 0.9, and for all SNPs in the dataset, minor allele frequency (MAF) ≥ 10%, missing genotype data rates (for SNPs and individuals) ≤ 5%, and Hardy–Weinberg equilibrium (HWE) *P*‐value > 1 × 10^−08^. All imputed SNPs included in the statistical analyses had an info score > 0.8. For simplicity, we refer to the genetic variants in this dataset as ‘SNPs’, even though the genotyping platform and imputation results contain other variant types, such as INDELs.

**Table 1:** Baseline characteristics of the SNP and CNV/INDEL analysis cohorts.

Variable	SNP analysis cohort (*n* = 505)		CNV/INDEL analysis cohort (*n* = 495^a^)	
	Number	%	Number	%
Age at diagnosis				
Median (range)	61.43 (20.70–75.01)	–	61.40 (20.70–75.01)	–
Sex				
Male	307	60.79	301	60.81
Female	198	39.21	194	39.19
Tumor location				
Colon	334	66.14	328	66.26
Rectum	171	33.86	167	33.74
Stage				
I	93	18.42	89	17.98
II	196	38.81	193	38.99
III	166	32.87	164	33.13
IV	50	9.90	49	9.90
Histology				
Nonmucinous	448	88.71	438	88.48
Mucinous	57	11.29	57	11.52
Grade				
Well/moderately differentiated	464	91.88	457	92.32
Poorly differentiated	37	7.33	34	6.87
Unknown	4	0.79	4	0.81
MSI status				
MSI‐L/MSS	431	85.35	421	85.05
MSI‐H	53	10.50	53	10.71
Unknown	21	4.16	21	4.24
Adjuvant chemotherapy treatment				
No	224	44.36	217	43.84
Yes	277	54.85	274	55.35
Unknown	4	0.79	4	0.81
Adjuvant radiotherapy treatment				
No	364	72.08	355	71.72
Yes	124	24.55	123	24.85
Unknown	17	3.37	17	3.43
Follow‐up time				
Median (range)	13.79 (0.38–19.00)	–	13.80 (0.38–19.00)	–
DSS status				
Death from other causes or alive	332	65.74	323	65.25
Death from colorectal cancer	99	19.60	99	20.00
Unknown	74	14.65	73	14.75
Death from other causes or alive (within 5 years)	407	80.59	398	80.40
Death from colorectal cancer (within 5 years)	62	12.28	62	12.53
Unknown (within 5 years)	36	7.13	35	7.10
RMFS status^b^				
Recurrence or metastasis (−)	331	72.75	322	72.20
Recurrence or metastasis (+)	124	27.25	124	27.80
Recurrence or metastasis (−) (within 5 years)	348	76.48	339	76.01
Recurrence or metastasis (+) (within 5 years)	105	23.08	105	23.54
Unknown (within 5 years)^c^	2	0.44	2	0.45

CNV, copy number variation; DSS, disease‐specific survival; INDEL, insertion/deletion; MSI, microsatellite instability; MSI‐H, microsatellite instability‐high; MSI‐L, microsatellite instability‐low; MSS, microsatellite stable; RMFS, recurrence/metastasis‐free survival; SNP, single nucleotide polymorphism.

^a^ Note that all 495 patients in the CNV/INDEL analysis cohort are also in the SNP analysis cohort with 505 patients.

^b^ Stage I–III patients only, total *n* = 455 in the SNP analysis cohort and total *n* = 446 in the CNV/INDEL analysis cohort.

^c^ ‘Unknowns’ appear because two patients had unknown survival time. Although they experienced recurrences/metastases, we do not know whether they had these events within the first 5 years postdiagnosis or after that.

In addition to the outcome measures examined, the SNP dataset largely differs from the dataset that we used in a previous genome‐wide association study [21] (due to the imputation that allowed us to obtain genotypes of additional variants and the use of longer follow‐up data in this study).

The second genetic dataset consists of a set of CNVs/INDELs (Table S1) [29]. The CNV/INDEL dataset (*n* = 3486) was previously obtained by our team [29] using a computational pipeline that included penncnv [43] and quantisnp [44] software. These analyses are described in detail in Werdyani *et al*. [29]. In short, MAP file and signal intensity data obtained by the Illumina^®^ Human Omni1_Quad_v1 genome‐wide SNP genotyping array (Log R ratio (LRR) and B allele frequency (BAF) measures) were used as input files to computationally predict the CNV/INDEL profiles using quantisnp [44] and penncnv [43] algorithms. These algorithms are designed to detect CNVs from the whole‐genome SNP genotyping platform data based on a hidden Markov model (HMM) [43, 44]. Prediction of the CNVs/INDELs by the quantisnp algorithm was performed using the signal intensity files of each patient using default parameters [44]. To detect the CNVs/INDELs by the penncnv algorithm, population frequency of B allele (PFB) and the GC‐model file for the Illumina^®^ Human Omni1_Quad_v1 platform were generated based on the hg19 genome coordinates [43]. An adjustment of genomic waviness was implemented [45, 46, 47], and calls were restricted to the autosomal chromosomes [48, 49]. Low‐quality CNV/INDEL calls were filtered out using the QC metrics provided by quantisnp and penncnv [50, 51, 52, 53]. We identified CNVs/INDELs that were called by both algorithms (the same copy number state (CN) and overlapped at least 50% of their sequences) using a custom Perl program [53, 54]. Of note, 84.3% of such variants had identical start and end positions. In other cases, overlapping variations were merged together [52]. Since detection of CNVs/INDELs in highly repetitive sequences results in high false‐positive calls (e.g., centromere and telomere regions, immunoglobulin and olfactory receptor (OR) gene regions [43, 55, 56]), variants that intersected at least one bp with these DNA regions were excluded from further analyses. Finally, to reduce the false‐positive calls, variants that overlapped (at least 50% of their sequences) with previously experimentally validated CNVs [57, 58, 59] (included in the Database of Genomic Variants (DGV) [60]) were identified. These CNVs/INDELs are considered to be most likely true variations and constituted the final list of CNVs/INDELs that were predicted with high confidence. DNA analysis showed a high concordance rate for homozygous deletions (CN state = 0). For further details, please see Werdyani *et al*. [29]. These high‐confidence CNV/INDEL data were available for 495 patients (Table 1). These 495 patients were also included in the SNP dataset cohort described above. 254 CNVs/INDELs (Table S1) that passed filtering based on having copy number state of 0 (i.e., homozygous deletion) in 10–90% in the patient cohort were analyzed. We had previously examined the associations of 106 of these CNVs/INDELs in the patient cohort with a different outcome measure defined based on a shorter follow‐up data [29].

### 2.3 SNP imputation

The 1000 Genomes Phase 3 data (downloaded from the impute2 website: https://mathgen.stats.ox.ac.uk/impute/1000GP_Phase3.html) were used as the reference panel data. These data include 2504 individuals and more than 80 million variants [41]. These individuals were individuals from different population groups, including Europeans. The impute2 developers recommend to use this inclusive reference panel because the imputation is often more accurate by using this panel than other smaller panels chosen by intuition (e.g., a panel with only Europeans; http://mathgen.stats.ox.ac.uk/impute/impute_v2.html). The impute2 program can automatically choose a ‘custom’ reference panel for each individual of interest from the inclusive reference data, and this has been proved to work in variety of populations, including the homogeneous isolates (http://mathgen.stats.ox.ac.uk/impute/impute_v2.html). The data on variants in the reference panel with 2504 individuals were released in NCBI build 37 (hg19) coordinates, which is the same version as our genotyped SNP data.

The methodology applied in this study includes two major steps: phasing and imputation. Before phasing, genotyped SNPs were aligned to the positive DNA strand (i.e., the same strand as in the reference data). For unambiguous SNPs (i.e., SNPs with the allele types A/G, A/C, T/G, or T/C), the strands were easy to define because the alleles would be the complementary ones if the genotyped strands were opposite of the reference strand. For example, a SNP with A/G alleles would be on the negative strand if the alleles of the same SNP in the reference data were T/C. As for the ambiguous SNPs (i.e., SNPs with alleles of A/T or C/G), similar to other studies [61, 62] we made use of the MAFs and reasoned that they would be similar between our data and the data of Europeans in the reference panel. Those ambiguous SNPs with MAFs larger or equal to 40% were excluded because it is difficult to determine their strands based on the MAF. The DNA strand of the ambiguous SNPs with MAFs < 40% was estimated by comparing their allele types to the data of Europeans in the reference data. If the minor alleles between the genotype data and the data of Europeans in the reference panel were the same, these SNPs were assumed to be on the same DNA strand. When the minor alleles were complementary to each other, then the ambiguous SNPs in the study data were assumed to be on the negative strand; these SNPs were then flipped to the positive strand by using plink (v1.07) [42]. Last, SNPs with different allele types compared to the reference SNPs and those SNPs existed in our data while not listed in the reference panel were excluded. A total of 7244 SNPs were excluded during this step. In the end, 803 918 SNPs remained in the dataset for imputation.

The software shapeit (v2.r837) [39] and impute2 (v2.3.2) [40] were used for phasing and imputation steps, respectively. Genotype dataset was first separated for each chromosome using plink (v1.07) [42], and then, phasing was performed for each chromosome as recommended in the shapeit tutorial (http://mathgen.stats.ox.ac.uk/genetics_software/shapeit/shapeit.html). During this step, the default or recommended parameters were used; ‐‐states parameter was set as its default value (100) and the effective size of 11 418 was used, which is the effective size recommended for Europeans by the developers of shapeit. The same value of effective size has been used in the genetically isolated Finland population for phasing [63]. shapeit has been reported to be able to phase populations with a wide spectrum of relatedness, including isolated populations [64].

The phased data for each chromosome were then used as the input for imputation. To do so, first, data from each chromosome were split into small segments as suggested by the tutorial provided by the impute2 program’s official website (https://mathgen.stats.ox.ac.uk/impute/impute_v2.html#ex2). Imputation requires a number of genotyped SNPs/segments to construct the possible haplotypes (https://mathgen.stats.ox.ac.uk/impute/impute2_overview.html; https://genome.sph.umich.edu/wiki/IMPUTE2:_1000_Genomes_Imputation_Cookbook). As recommended (http://mathgen.stats.ox.ac.uk/impute/impute_v2.html), in this study, each chromosome was initially split into 5 Mb segments starting from the telomeres at the p‐arm of each chromosome. Each segment should contain at least 200 SNPs for imputation, as suggested by other researchers (https://genome.sph.umich.edu/wiki/IMPUTE2:_1000_Genomes_Imputation_Cookbook). If this was not the case, then such segments were merged with a nearby (i.e., preceding) segment on the same chromosomal arm. Note that telomere and centromere segments may contain < 200 SNPs as genotyping these genomic regions are problematic because of their repetitive sequences [65]. As per the segments that overlap with the centromeres, we made sure that the boundaries of the segments on the p‐arm were extended to the end of each of the centromere. This also means that the start position of the next segment on the q‐arm was right after the end of the centromere. If these latter segments included < 200 SNPs, they were merged with the successive segment on the q‐arm. The p‐arms of chr 13, 14, 15, 21, and 22 did not have enough genotyped SNPs (*n* = 4 for chr 21 and *n* = 0 for other chrs)—so no imputation have been performed for these chromosomal arms. In the end, 548 final chromosomal segments from 22 chromosomes were generated. After this step, –int parameter was used in impute2 to conduct the imputations within each specific chromosomal segment (e.g., ‐int 5 000 001 10 000 000 defines a segment between 5 000 001 bp and 10 000 000 bp). As for segments that were larger than 7 Mb (e.g., merged segments), an additional command ‐allow_large_regions was used for imputation. The parameter –Ne was set as 20 000 because impute2 developers recommend this number (https://mathgen.stats.ox.ac.uk/impute/impute_v2.html#ex2). Other parameters were set at default values. Also, to achieve high‐quality imputation for SNPs at ends of each segment, by default a buffer region of 250 Kb was automatically assigned to ends of the segments.

After imputation, a number of segment‐specific output files were generated for each chromosome. The data in these files were then combined together to create files (i.e., chromosome output files) that contain the imputation data per each chromosome.

The data in the chromosome output file were then converted to PLINK PED files using gtool (v0.7.5) (http://www.well.ox.ac.uk/~cfreeman/software/gwas/gtool.html). In this process, post‐QC measures were also implemented. For example, SNPs with an info score > 0.7 [62, 66, 67] and a maximum probability of the imputed genotypes larger than 90% [68] were included in the final PED files. Info score is an important indicator used to estimate imputation certainty. The closer this score is to 1.0, the higher the certainty about the imputation (http://mathgen.stats.ox.ac.uk/impute/impute_v2.html ) [69]. The maximum probability of the imputed genotypes of a given SNP defines the most possible genotype of that SNP. For example, a SNP with the allele type of A/G can have three possible genotypes AA, AG, and GG. After imputation, each genotype in an individual is given a ‘probability’ value by impute2, say 0.05, 0.08, and 0.87. The maximum probability for the SNP genotype in this case is 0.87 (87%), which means the most likely genotype of the individual is GG.

More than 38 million variants were imputed with an info score > 0. The range of the concordance rate of imputations was 94–99.9% with a median of 98.7%. The concordance rate was estimated by comparing the genotypes of the known variants to their imputed genotypes and was done automatically by the impute2 program as part of its imputation process. In addition, twenty‐two ambiguous SNPs that were excluded prior to phasing (one SNP per chromosome) were randomly selected and the concordance between the real and imputed genotypes was examined. The result of this examination showed that only 37 discrepancies were found among the 11 110 genotypes (22 SNPs * 505 individuals), which accounts for a concordance rate of 99.7%. Note that in the dataset, the genotyped variants would have an info score and probability of 1.0. Thus, at the end the total number of variants (including genotyped ones and imputed ones) satisfying the info score and probability thresholds was 13 974 610.

The distribution of info scores for all imputed variants is shown in Fig. S1. Most variants had either very low or very high info scores. Fig. S2 shows the relationship between the average info score and the MAF of the variants. Among the ˜ 38 million imputed variants, the majority of the variants were quite rare (MAFs < 0.02), whereas ˜ 6.3 million variants (˜ 1/6) were common (i.e., had MAF ≥ 0.05) (Fig. S2). The info scores increased as the MAFs increased, as expected [62], and were particularly low for the variants with MAFs < 0.02. The average info scores for the rest of the variants (MAFs ≥ 0.02) were high (> 0.8) (Fig. S2). As shown in Fig. S3, the majority of the common SNPs (MAFs ≥ 0.05) had very high info scores, which means these variants had high imputation quality. To be more specific about this point, 6 163 520 common and imputed variants had an info score > 0.7, which accounts for 97.9% of all variants with MAF ≥ 0.05. By comparing Figs S1 and S3, we can say that almost all variants with low info scores were variants with MAF < 0.05 (the bars representing the number of variants at the low info sections of Fig. S1 almost disappeared in Fig. S3). In this study, we limit our analyses to 4 711 309 SNPs that satisfied the inclusion criteria (see Patient cohort and clinical and genetic data).

### 2.4 Statistical analyses

#### 2.4.1 Correlation among the variables

LD *r* ^2^ values were calculated for genetic variants using plink v1.07 [42]. Pair‐wise Pearson correlation coefficient (*r*) values were calculated for baseline variables (Table S2), which suggested that no collinearity (*r* < 0.8) existed among these variables.

#### 2.4.2 Outcome measures

The outcome measures are disease‐specific survival (DSS) and recurrence/metastasis‐free survival (RMFS). Endpoint events in these outcome measures are death from colorectal cancer and local recurrence or distant metastasis, respectively. DSS and RMFS times are calculated as the times from the date of diagnosis till the date of the occurrence/diagnosis of these events or the date of last alive contact. DSS was examined for stage I‐IV patients, and RMFS was analyzed for stage I‐III patients only (Table 1).

#### 2.4.3 Survival analysis

Univariate Cox models were fitted for 4 711 309 SNPs for both outcome measures separately. The proportional hazards (PH) assumption was tested under the univariate Cox models using the cox.zph function of the survival package [70] in r [71]. SNPs that satisfied the PH assumption (*P*‐value of the PH assumption test ≥ 0.05) were then checked for their Cox regression *P*‐values. Those with *P*‐values < 5 × 10^−06^ were retained for multivariate analysis (Figs S4 and S5). On the other hand, SNPs that violated the PH assumption (i.e., variants with possible time‐varying associations) were refitted in univariate piece‐wise/change‐point Cox PH regression models [72, 73] with a time point of 5 years as the cutoff time point. Five years was chosen as the time point to help practically fit a large number of SNPs that violate the PH assumption while also providing a clinically meaningful time point. PH assumption was then checked for these SNPs before and after the 5 years cutoff time point. Those that satisfied the PH assumption at both time intervals and had Cox regression *P*‐values < 5 × 10^−06^ before and/or after 5 years postdiagnosis were selected for multivariate analysis (Table S3). Select Manhattan, regional, and QQ plots are depicted in Figs S4–S9. The genomic regions/loci with independent association signals are defined as ± 500 kb of the identified variants with the smallest *P*‐values (i.e., index variant), while also considering the LD information (other identified variants in these regions should have *r* ^2^ ≥ 0.8 with the index variants).

Covariates used to adjust the associations of SNPs in multivariate models were identified through the process of baseline model construction. In short, baseline models were constructed using the backward selection method (considering the clinical variables shown in Table 1) as described in Yu *et al*. [34], followed by force entering the adjuvant chemotherapy and adjuvant radiotherapy statuses. During the process of baseline model construction, covariates that violated the PH assumption were assigned proper cutoff time points, which ensured that they satisfied the PH assumption within the time intervals defined by these cutoff time points. The method to identify the cutoff time points for variables that violate the PH assumption in Yu *et al*. [34] was used. In short, the proper cutoff time point for a given clinical variable that violated the proportional hazards (PH) assumption was identified during the backward selection procedure, as follows: (a) Time points (ranged from 0.5 years to 18.5 years, with increments of 0.5 years) were used for the variable to fit Cox models; (b) the log partial likelihood values of models for each time point were obtained; and (c) the PH assumption for the variable before and after the cutoff time points in these models was checked. The proper cutoff time point was determined to be the one that makes (a) the corresponding model with the largest log partial likelihood value and (b) the PH assumption being satisfied both before and after the cutoff time point. Variables that were not significant in the models (Cox regression *P*‐values > 0.05) were removed one by one during the selection process. Final baseline models included significant clinical variables (Cox regression *P*‐value < 0.05) as well as the force‐entered treatment related covariates, which also satisfied the PH assumption (*P*‐value of PH assumption test ≥ 0.05). For further details about this approach, please see Yu *et al*. [34]. In the end, tumor location (with a cutoff time point of 6 years), disease stage, microsatellite instability (MSI) status, adjuvant chemotherapy, and adjuvant radiotherapy (with a cutoff time point of 7 years) were remained in the final baseline model for DSS. For RMFS analysis, tumor location (with a cutoff time point of 3 years), disease stage, and adjuvant chemotherapy and radiotherapy treatments were included in the final baseline model.

These baseline variables were then used as covariates in multivariate analysis adjusting the association of variants with survival outcomes. Principal component analysis (PCA) in the patient cohort did not indicate population stratification (the top principal component accounted for merely 0.3% of the total variance); hence, principal components obtained from the genetic data were not included as covariates. At the time of fitting the multivariate models (i.e., when SNPs were entered into the baseline model one by one), the PH assumption was checked again for all variables in these models, including the tested genetic variants and clinical covariates. If variants violated the PH assumption, then they were analyzed in refitted multivariate Cox models with 5 years entered as the cutoff time point. If the covariates violated the PH assumption, then their proper cutoff point(s) were identified/re‐identified, followed by refitting the multivariate models as described by Yu *et al*. [34] (note that none of such models included variants that reached the genome‐wide significance level). The final multivariate Cox models are the ones with the PH assumption satisfied for all variables. Hazard ratios (HRs) and 95% confidence intervals (CIs) were obtained from the multivariate Cox models.

SNPs in this study were examined under additive, dominant, and recessive genetic models. We included recessive model in order not to miss potential associations; however, results should be taken with caution because of the rarity of the homozygous genotypes. Variants with Cox regression *P*‐values < 5 × 10^−08^ (either during the entire follow‐up [i.e., with no time‐varying associations] or before and/or after 5 years postdiagnosis [i.e., with time‐varying associations]) were considered to be the variants that were significantly associated with the survival outcome.

Statistical analysis of the CNV/INDEL dataset followed the same analysis procedure as the SNP dataset. During the statistical analyses, patients with homozygous deletions were compared with the patients with other copy number states (i.e., ≥ 1 copy of the variant).

The empirical power (based on 10 000 simulation replicates) was calculated using the survsnp package [74] in r [71]. This study has at least 80% power to detect effect sizes of 3.2, 3.6, and 18.4 (in DSS analysis) and 3.0, 3.4, and 16.8 (in RMFS analysis) under the additive, dominant, and recessive models, respectively, for variants with a MAF of 10%. Generally, increased power is expected as MAF increases. We expect the same power for the first interval (i.e., the first 5 years postdiagnosis), but less power for the second interval, as the number of events is less in that time period.

Statistical analyses were performed using r ver. 3.5.0 [71] unless otherwise specified. Kaplan–Meier curves, Manhattan, and QQ plots were generated using the survival [70] and qqman [75] packages in r [71], respectively. Regional plots were created using software locuszoom [76].

### 2.5 Validating associations in the TCGA cohort

White (excluding Hispanics/Latinos) colorectal cancer patients with primary tumors were selected. Clinical and outcome data were downloaded from the Genomic Data Commons (GDC) data portal [77] (https://portal.gdc.cancer.gov/; nationwidechildrens.org_clinical_patient_coad.txt, nationwidechildrens.org_clinical_patient_read.txt, nationwidechildrens.org_auxiliary_coad.txt, nationwidechildrens.org_auxiliary_read.txt) (on Dec 13 – 14, 2020) and a study published in 2018 [78], respectively. Germline genetic data of patients (obtained from blood) were obtained from birdseed files in the GDC Legacy Archive [77] (on Nov 16, 2020). High‐confidence genotype calls (birdseed confidence value < 0.1) of SNPs were extracted, and those genotypes with low‐confidence calls were set as ‘missing’. As a result, clinical and genetic data were available for 266 patients. Among the 266 patients, four were removed because they either had a high heterozygosity rate or were possible relatives, population outliers, or non‐European. The final TCGA cohort consisted of 262 unrelated colorectal cancer patients (Table S4). These data and procedures are described in detail as follows: Germline genetic data (Affymetrix genome‐wide human array 6.0) of colorectal cancer patients (COAD and READ) were obtained from birdseed files (one file per patient) from the GDC Legacy Archive (https://portal.gdc.cancer.gov/legacy‐archive/search/f). SNP data from different birdseed files were combined and converted to a single plink PED/MAP file through the following steps [79]: (a) Genotyping calls (in the format of allele counts 0, 1, or 2) from birdseed files were first assigned as ‘missing’ for low‐confidence SNPs (confidence value ≥ 0.1); (b) information of SNPs’ genotyping calls from birdseed files was then combined; (c) probe IDs were replaced with rs numbers for all SNPs based on the information in the annotation file of the Affymetrix genome‐wide human SNP array 6.0; (d) duplicated SNPs (*n* = 2) were removed (the one with more missing data); (e) duplicated samples (*n* = 4) were removed (the one with more missing data); (f) allele counts were converted to genotypes composed of A, T, C, and G; (g) additional required information was added to form the final PED‐formatted file (sex information was derived from GDC clinical data; phenotype was assigned to 2 [i.e., affected; colorectal cancer patients]; paternal and maternal IDs were assigned to 0; and family IDs were assigned the same as individual IDs); and (h) the MAP file was constructed based on the Affymetrix annotation file. In the end, 266 patients and 906 598 SNPs were included in the PLINK PED/MAP file. In this 266 patients cohort, patients were excluded if they (a) have any mismatched sex information (between sex information in the clinical data and the sex information imputed by PLINK from genetic data; *n* = 0); (b) have genotyping call rate < 5% (*n* = 0); (c) have a high heterozygosity rate (out of 6 SD) (*n* = 1); (d) are duplications or possible relatives (identity‐by‐state [IBS] PI_HAT score > 0.125) [21] (*n* = 1); (e) are population outliers (the minimum *Z* score of individual’s IBS distances to five nearest neighbors < −4) [42, 80] (*n* = 1); (f) are possible non‐European descendants (comparing to the 1000 Genomes phase3 data in the multidimensional scaling [MDS] plot which was created based on the usage of the ‐‐genome and ‐‐mds‐plot flags in plink1.9 [81]) (*n* = 1). After these steps, 262 colorectal cancer patients remained in the cohort.

The genetic data of the 262 patients were then used for principal component analysis (PCA) using plink1.9 [81]. SNPs used for PCA were those that (a) locate on autosomal chromosomes, (b) have MAFs ≥ 1%, (c) have missing call rates < 5%, (d) have HWE *P*‐values ≥ 1 × 10^−06^, (e) locate outside the long‐LD regions [82], and (f) are independent SNPs (SNPs remained after pruning; pair‐wise LD *r* ^2^ < 0.2) [83]. In the end, 115 051 SNPs of the 262 patients were used for PCA. The top PC (Fig. S10) accounts for 0.9% of total variance.

Genotypes for the SNP identified in the patient cohort (*WBP11*‐rs7314075) were not available in this cohort, but genotype data were available for six SNPs (rs11056174, rs2041909, rs2041908, rs6488711, rs2241221, and rs11835363) that are in high LD with it (*r* ^2^ > 0.8 based on the European data (EUR) in Haploreg 4.1 database [84]). Genotype data of these SNPs were used to examine their associations with DSS in multivariate Cox models with disease stage, tumor location, MSI status, and the top principal component as the covariates (Fig. S10). In all Cox models, PH assumption was checked and satisfied for both clinical and genetic variables.

Among the 12 SNPs in three loci identified under the recessive genetic model in DSS analysis and their 28 high‐LD SNPs, one identified SNP rs12757197 (also named as kgp2690683 in the NFCCR cohort) and three high‐LD SNPs (rs358347, rs357167, and rs165269) were included in the TCGA dataset. However, these SNPs either had no genotypes with double minor alleles (rs12757197) or had no reliable effect estimations (rs358347, rs357167, and rs165269 had ‘infinity’ appeared in their upper limit of the 95% confidence interval) in the analysis using the TCGA data.

### 2.6 Examining the associations of CMS with SNPs in high LD with rs7314075 and *WBP11* expression levels in the TCGA dataset

As per the recommendation of one of the reviewers, we also checked the associations between the genotypes of the SNPs in high LD with *WBP11*‐rs7314075 as well as the *WBP11* tumor gene expression levels with tumor consensus molecular subtypes (CMS) in the TCGA dataset. *WBP11* expression data were downloaded from the UCSC Xena [85], and tumor CMS information was obtained from a study published in 2015 [86]. Fisher’s exact test was utilized for testing the association of SNP genotypes with the CMS classifications, and Kruskal–Wallis test was used to examine the associations of *WBP11* gene expression levels and CMS classifications (ANOVA was not used because the normality assumption was violated). When a significant association was detected by the Kruskal–Wallis test, further pair‐wise comparison was performed using Dunn’s test to see which two CMS groups have different *WBP11* expression levels.

### 2.7 Bioinformatics analyses

The functional consequences of the SNPs identified (and SNPs that are in high LD with them according to the Haploreg database v4.1 [84], based on the European population) were checked in the RegulomeDB database (v2.0) [87] and GTEx (data release v8) [88] (GTEx had data for colon, but not rectum tissues). Expression levels of genes in tumors and adjacent normal tissues (noted as ‘solid tissue normal’ in TCGA) were explored in UCSC Xena [85] using the colorectal tissue data from TCGA [89]. The gnomAD database [90] was used to search for SNP frequencies in different populations. Official gene names and basic definitions of gene functions were retrieved from Gene Entrez [91].

## 3 Results

### 3.1 Associations between SNPs and survival outcomes

In this study, we examined 505 and 495 Caucasian patients from Newfoundland, Canada, in the SNP and CNV analysis parts, respectively. Patients were followed up to 19 years. During this period, 99 patients had died from colorectal cancer and 124 patients had experienced recurrence and/or metastasis (Table 1).

Associations (*P* < 5×10^−08^) that are detected for disease‐specific survival (DSS) and recurrence/metastasis‐free survival (RMFS) in multivariable analyses are shown in Table 2 and Tables S5 and S6.

**Table 2:** rs7314075 that is significantly associated with disease‐specific survival (DSS) in multivariate analysis under the *dominant* and *additive* genetic models.

Chr	Pos	Minor/major allele	MAF	Variant type	Info score	Genetic model	HR (95% CI)^a^	*P*‐value	*P*‐value of the PH assumption test	Located region^b^
12	14945417	A/G	0.14	Imputed	0.964	Dominant	3.36 (2.18, 5.16)	3.27 × 10^−8^	0.96	Intron of *WBP11*
						Additive	2.65 (1.88, 3.75)	3.24 × 10^−8^	0.63	

Models are adjusted for MSI status, disease stage, tumor location (6 years as the cutoff time point), adjuvant chemotherapy, and radiotherapy statuses (7 years as the cutoff time point for adjuvant radiotherapy).

Chr, chromosome; CI, confidence interval; HR, hazard ratio; MAF, minor allele frequency; PH, proportional hazard; Pos, position.

^a^ Hazard ratio was estimated under the dominant genetic model for [AA+AB] vs BB and under the additive genetic model for AA vs AB vs BB, where A is the minor allele and B is the major allele.

^b^ Gene annotation is obtained from the UCSC database [96] (‘UCSC genes’ from the UCSC browser [GRCh37/hg19]).

#### 3.1.1 Associations with constant HRs

**Fig. 1:**
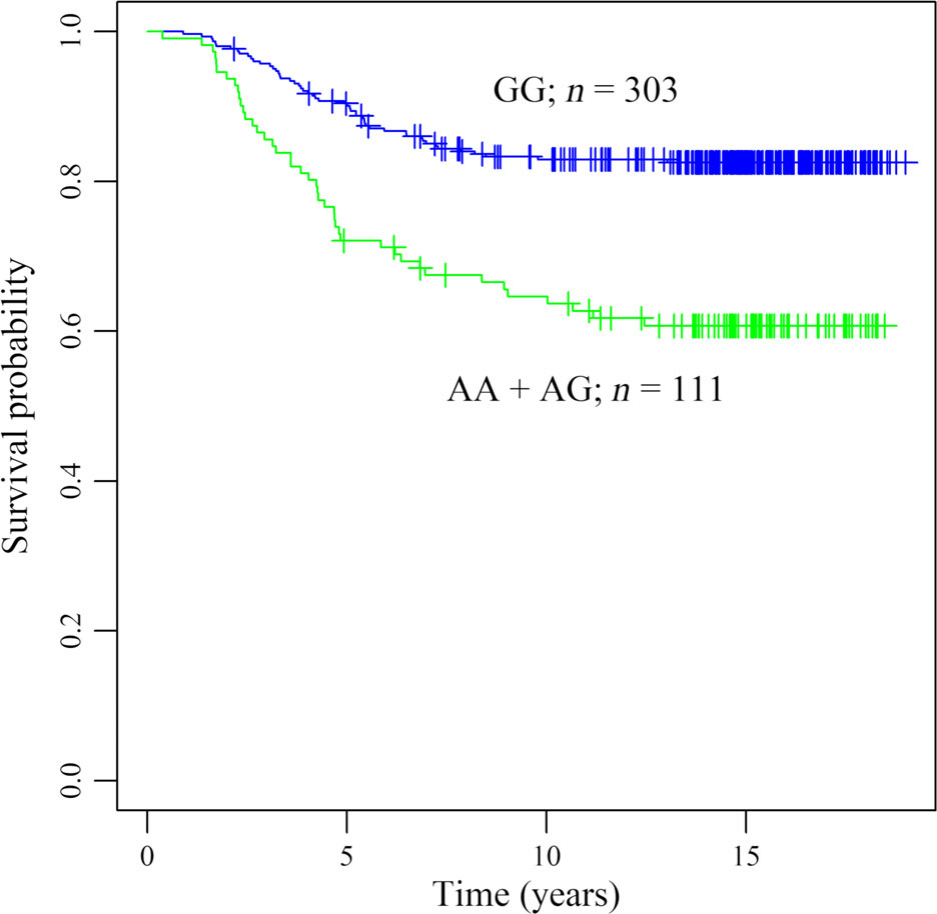
Kaplan–Meier curves of rs7314075 in the disease‐specific survival (DSS) analysis under the *dominant* genetic model. The *P*‐value of the log‐rank test is 2 × 10^−06^.

After adjustment for clinical covariates, one common SNP that locates in an intron of *WBP11* (rs7314075) was significantly associated with the risk of death from colorectal cancer under both dominant (HR = 3.36; *P*‐value = 3.27 × 10^−08^) and additive (HR = 2.65; *P*‐value = 3.24 × 10^−08^) genetic models (Table 2). Under the dominant genetic model (Fig. 1), patients with AA or AG genotype had more than three times of the risk of death from colorectal cancer compared to patients with GG genotype. Under the additive genetic model, in line with the results of the dominant genetic model, risk of death from colorectal cancer increased more than 1.5 folds as per A allele (i.e., the minor allele). With regard to SNPs examined under the dominant and additive models in the RMFS analysis, none of them reached significant *P*‐values in the multivariate analysis. Top SNPs with suggestive associations for these genetic models are shown in Table S7.

Under the recessive genetic model, associations were detected in multivariate analyses for 13 genomic regions (a total of 12 SNPs from three genomic loci in DSS and 56 SNPs from 10 loci in RMFS analyses) that passed the genome‐wide significance level of 5 × 10^−08^ (*P*‐values 10^−08^–10^−12^) (Tables S5 and S6). Some of these variants were located in genes (Tables S5 and S6). Since many of these associations included small numbers of minor allele homozygous genotypes, these results should be approached with caution.

#### 3.1.2 Time‐varying associations

Interestingly, three variants from two different genomic loci (chromosomes 2 and 12: rs200143895, rs11064732, rs817090) had time‐varying associations with RMFS under the recessive model after adjustment for clinical covariates. These variants were associated with the risk of recurrence/metastasis only after 5 years postdiagnosis (Table S6).

No SNPs with time‐varying associations were detected in other models examined in multivariate analysis.

### 3.2 Examining the association of *WBP11*‐rs7314075 with DSS in the TCGA cohort

*WBP11*‐rs7304075 itself was not included in the TCGA genetic data, but there were six SNPs (Table 3) that were in high linkage disequilibrium (LD) (*r* ^2^ > 0.8) with it in this dataset. These SNPs were analyzed for their associations with DSS in the TCGA colorectal cancer cohort. Four SNPs (rs11056174, rs2041909, rs6488711, and rs2241221) were significantly associated with the risk of death from colorectal cancer under both dominant and additive genetic models (adjusted for tumor location, disease stage, MSI status, and the top principal component) (Table 3). Consistent with the results obtained in our patient cohort (Table 2), genotypes containing the minor alleles of these SNPs were associated with an increased risk of outcome in the TCGA patient cohort (HRs = 2.93–3.00 under the dominant genetic model; HR–2.32–2.39 under the additive model) (Table 3).

**Table 3:** Associations between SNPs in high LD with rs7314075 and disease‐specific survival (DSS) in multivariate analysis in the TCGA dataset under the *dominant* and *additive* genetic models.

Genetic model	SNP	Chr	Pos	Minor/major allele	MAF	HR (95% CI)^a^	*P*‐value	*P*‐value of the PH assumption test
Dominant	rs11056174	12	14909977	T/C	0.14	2.94 (1.20, 7.20)	**0.018**	0.56
	rs2041909	12	14915409	C/T	0.14	3.00 (1.23, 7.32)	**0.016**	0.58
	rs2041908	12	14916150	G/A	0.14	2.32 (0.96, 5.65)	0.063	0.73
	rs6488711	12	14933216	T/C	0.14	2.93 (1.20, 7.17)	**0.018**	0.56
	rs2241221	12	14959391	C/T	0.16	2.97 (1.23, 7.16)	**0.015**	0.47
	rs11835363	12	14982700	C/T	0.16	2.42 (1.00, 5.88)	0.050	0.23
Additive	rs11056174	12	14909977	T/C	0.14	2.35 (1.05, 5.29)	**0.038**	0.81
	rs2041909	12	14915409	C/T	0.14	2.38 (1.06, 5.32)	**0.035**	0.85
	rs2041908	12	14916150	G/A	0.14	1.96 (0.87, 4.44)	0.106	0.92
	rs6488711	12	14933216	T/C	0.14	2.32 (1.03, 5.20)	**0.041**	0.79
	rs2241221	12	14959391	C/T	0.16	2.39 (1.08, 5.31)	**0.032**	0.72
	rs11835363	12	14982700	C/T	0.16	2.01 (0.90, 4.50)	0.091	0.39

Models are adjusted for MSI status, disease stage, tumor location, and the top principal component. Bolded values are *P*‐values < 0.05, indicating significant associations between variants and DSS.

Chr, chromosome; CI, confidence interval; HR, hazard ratio; MAF, minor allele frequency; PH, proportional hazard; Pos, position.

^a^ Hazard ratio was estimated under the dominant genetic model for [AA+AB] vs BB and under the additive genetic model for AA vs AB vs BB, where A is the minor allele and B is the major allele.

### 3.3 Functional roles of SNPs

**Fig. 2:**
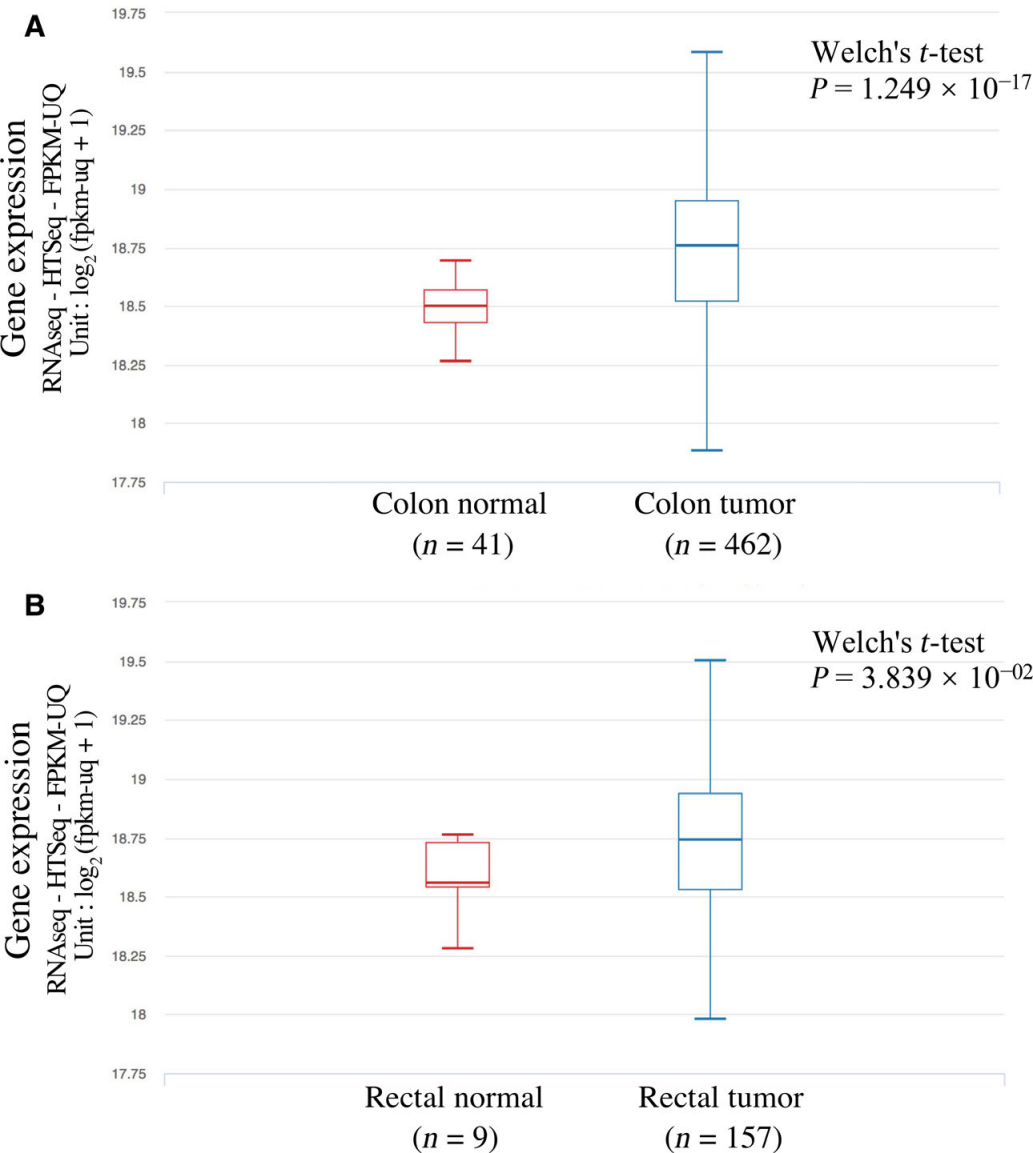
Expression level of *WBP11* in colorectal tumors and normal tissues. Analysis was done in UCSC Xena [85] using the GDC TCGA COAD and READ data. In both datasets, primary tumors and adjacent normal tissues (noted as ‘solid tissue normal’ in TCGA data) were selected (recurrent and metastatic tumors were excluded). Then, only tumors and normal tissues with their anatomical sites noted as colon (in COAD) and rectum and rectosigmoid junction (in READ) were analyzed. (A) *WBP11* expression in colon tumors and normal tissues from the TCGA COAD cohort; (B) *WBP11* expression in rectal tumors and normal tissues from the TCGA READ cohort. Expression of *WBP11* is significantly higher in colon and rectum tumors compared to normal tissues. The number of patients in the colon and rectum tumor datasets is larger than those in the normal tissue datasets. This may explain why the gene expression levels in tumors have a higher variance compared to that in the normal tissues.

We explored the potential functional features of *WBP11*‐rs7314075 and its highly linked SNPs. According to Haplogreg [84], there were 38 SNPs that were highly linked with the *WBP11*‐rs7314075. Two of these highly linked SNPs (rs2241221 and rs11056174) were cis‐eQTLs (i.e., located within ± 1 Mb region of the transcription start sites of the associated genes) according to RegulomeDB [87] (Table 4). These SNPs were associated with the expression level of *ERP27* in monocytes. Comparison of gene expression levels using the TCGA data showed that the expression levels of *ERP27* and *WBP11* were higher in the colon and rectal tumors than in adjacent normal tissues (the ‘solid tissue normal’ in TCGA data) (Fig. 2 and Fig. S11).

**Table 4:** Variants that are in high LD with *WBP11*‐rs7314075 that are eQTLs.

Outcome—genetic model	rs ID	eQTL‐associated gene (tissue)—RegulomeDB^a^	eQTL‐associated gene (tissue)—GTEx^a^
DSS‐dominant/ additive	rs2241221	*FLJ32115/ERP27* (monocyte)	–
DSS‐dominant/ additive	rs11056174	*FLJ32115/ERP27* (monocyte)	–

DSS, disease‐specific survival; eQTL, expression quantitative trait locus; SNP, single nucleotide polymorphism.

^a^ Variants that are in high LD with *WBP11*‐rs7314075 (retrieved from Haploreg [84]) were explored in RegulomeDB [87] and GTEx [88]. Note that GTEx data were for colon tissue, as it has no data for rectal tissue. The eQTLs are all cis‐eQTLs that locate within ± 1 Mb of the transcription start sites of the genes shown in the table.

The three variants with time‐varying associations and their high‐LD SNPs were also examined, but none of them were found to be eQTLs. Other eQTLs identified in the recessive model analyses are shown in Table S8.

### 3.4 Examining the associations of high‐LD SNP genotypes and *WBP11* expression levels with CMS in the TCGA dataset

A nominal association was detected between rs2241221 and CMS (Fisher’s exact test *P*‐value = 0.052). Additionally, a significant association was found between *WBP11* expression levels and CMS (Kruskal–Wallis test *P*‐value = 9.66 × 10^−07^). Pair‐wise comparisons further showed that the expression levels of *WBP11* were different between CMS1, CMS2, and CMS4 in the TCGA dataset (Table S9).

### 3.5 Associations between CNVs/INDELs and survival outcomes

None of the CNVs/INDELs reached the *P*‐value threshold of 5 × 10^−06^ in the univariate analyses, therefore, were not selected for multivariable analyses. We show the top three CNVs/INDELs identified in the DSS and RMFS analyses in Table S10.

## 4 Discussion

We investigated the associations of a genome‐wide set of common SNPs and 254 CNVs/INDELs with time to death from colorectal cancer (DSS) and time to recurrence/metastasis (RMFS) in a colorectal cancer patient cohort with a long follow‐up. As a result, we identified one common SNP, *WBP11*‐rs7314075, that was significantly associated with DSS when adjusted for clinical factors (3.27 × 10^−08^ for dominant model, and 3.24 × 10^−08^ for additive model). A set of highly linked SNPs with *WBP11*‐rs7314075 were also associated with DSS in the TCGA patient cohort. This is one of the first replicated GWAS findings in colorectal cancer. This variant and the SNPs that are in high LD with them have not been reported in other GWASs [21, 22, 23, 24, 25, 26] and the candidate gene/pathway studies examining the colorectal cancer outcomes (based on the dbCPCO database [92]). Hence, these SNPs are novel candidate prognostic markers in colorectal cancer. In addition, we also identified fifteen genomic loci that were associated with clinical outcomes under the recessive model and they require validation in other independent cohorts. Interestingly, three variants in two genomic loci showed time‐varying associations; they predicted the outcome risk after 5 years, but not prior to this time point (i.e., candidate markers of late local/distant recurrent disease). To our knowledge, these variants are the first variants that can predict late recurrent disease in colorectal cancer. On the other hand, in contrast to SNPs, there were no associations of common CNVs/INDELs with the clinical outcomes examined. To our knowledge, it is one of the few GWASs examining colorectal cancer outcomes, the first GWAS that examines the germline sets of both SNP and CNVs/INDELs in the same patient cohort, and the most comprehensive study examining the time‐varying associations of genetic markers with clinical outcomes in colorectal cancer. Overall, with its comprehensive and unique study design, analysis, and results, this study significantly advances the prognostic research in colorectal cancer and expands the knowledge on the relationship of genetic variants with patient outcomes.

### 4.1 Associations with constant HRs (i.e., with proportional hazards)

One common SNP (rs7314075) was associated with DSS under both dominant and additive genetic models. Further investigations in the TCGA (COAD and READ) patient dataset strengthened our confidence in this association. Rs7314075 has a MAF of 14% in the patient cohort and locates in the 8^th^ intron of *WBP11*. *WBP11* encodes a protein that is involved in mRNA splicing [93]. Interestingly, a study on gastric cancer found that inhibiting *WBP11* expression results in the suppression of β‐catenin and thus suppression of proliferation and migration of tumor cells [94]. β‐catenin is a key component of WNT signaling pathway, which is involved in tumorigenesis and disease progress in colorectal cancer [95]. In line with the findings in gastric cancer [94], analysis of the TCGA data showed that the expression levels of *WBP11* in colon and rectum tumors were higher than in adjacent normal tissues. Also, the tumor *WBP11* expression levels were associated with CMS in the TCGA dataset, which is a gene expression‐based classification system and has been reported to have associations with outcomes in colorectal cancer [86]. These findings suggest a possible role of *WBP11* in colorectal cancer that needs to be examined further. According to RegulomeDB [87], there are two SNPs (that are in high LD with rs7314075) that are annotated as eQTLs in monocytes: rs2241221 and rs11056174. Interestingly, for both eQTLs, the target gene is identified as *FLJ32115/ERP27*. *ERP27* codes for an endoplasmic reticulum protein. An analysis of the TCGA data showed that, similar to *WBP11,* this gene has higher expression levels in colorectal tumors compared to nontumor samples (Fig. S11). Overall, findings by this study and existing literature suggest a possible biological relationship of *WBP11* with disease outcomes in colorectal cancer, and the *ERP27* gene can be an interest for future studies.

The remaining associations with DSS and RMFS were detected under the recessive genetic model and included variants from three and 10 genomic loci, respectively. While genotypes that are associated with outcomes are relatively rare, these SNPs/loci are worth examining in future studies with larger cohort sizes.

### 4.2 Time‐varying associations

Variants in two separate genomic regions were identified to have time‐varying associations (i.e., nonproportional hazards) in the RMFS analysis. These genetic markers were able to distinguish between patients with different outcome risk in the long term (after 5 years postdiagnosis). Minor allele homozygous genotypes of these SNPs predicted shorter RMFS times. According to the gnomAD database [90], the MAF of one of these SNPs (rs817090) is much higher in the African (30%) and Ashkenazi Jewish (18%) populations than Europeans. Therefore, it is possible that this SNP may predict the outcome risk of a higher number of colorectal cancer patients from these populations. All three variants are located in intergenic regions, and according to RegulomeDB [87] and GTEx [88], there is no strong evidence supporting potential regulatory functions. Similar results were obtained for the SNPs that are in high LD with them. These findings suggest that further studies are needed to elucidate the biological mechanisms that can explain these SNPs’ associations with the recurrent colorectal cancer in the long term.

Our study significantly contributes to the scientific knowledge on prognostic markers with time‐varying associations. This kind of markers are understudied in colorectal cancer [24, 29, 34, 35]. Since such variables may be missed by traditional analyses, application of appropriate statistical methods, as we have done in this study, is important to detect these variants. Additionally, such markers can provide unique clinical information (e.g., the time‐periods of high outcome risk), they can be quite useful in the clinic management of patients. Research into variants with time‐varying associations, therefore, should be encouraged. Should the time‐varying associations we detected be replicated in independent cohorts, these markers may be used to predict the colorectal cancer patients with a risk of recurrent disease after 5 years. Since clinic surveillance of patients for disease outcomes normally does not continue beyond the first 5 years, such information can be important to identify the patients who have high risk in the long term. This in turn can facilitate effective surveillance and clinical management of the patients at risk, with an anticipated improvement of their long‐term disease outcomes. We hope that our study will inspire more studies specifically looking for this clinically important type of prognostic markers.

### 4.3 Strengths and limitations

This study included common genetic variants, leaving rare variants to be investigated by further studies. We report associations, which are not the same as causation—this should be kept in mind while interpreting our results. We may have missed associations of rare variants and rare genotypes (especially in recessive genetic model analyses) or associations with small effects. Also, while we used a conservative *P*‐value threshold to control type I errors, we cannot rule out the possibility of false‐positive findings. Therefore, findings of this study need to be replicated in other colorectal cancer cohorts prior to any clinic utility can be established. In this study, 5 years was chosen as the cutoff time point in survival analysis of the variants that violated the PH assumption. This time point helps define simple and clinically meaningful models. However, there can be variants that have their cutoff time points other than 5 years; such variants can be an interest for future studies. The patient cohort has up to 19 years of follow‐up. To our knowledge, this is one of the longest follow‐up data in colorectal cancer, which allowed us to examine the time‐varying associations, particularly those that appear after the initial 5 years. Also, this study investigated different types of genetic variants (i.e., SNPs, CNVs/INDELs) in the same colorectal cancer cohort. This allowed us to have a comprehensive view of the relationships between genetic variants and survival outcomes in colorectal cancer. In addition, this study assumed no specific genetic model for the tested SNPs and included analyses under the three main genetic models. Such a comprehensive examination should have limited the possibility of missing SNPs with potential prognostic associations. We also detected the association of a set of SNPs that are highly linked with *WBP11*‐rs7314075 in the TCGA colorectal cancer cohort dataset, increasing our confidence in the association of this SNP with DSS. Last, we made sure that all variables in Cox models satisfied the PH assumption, which increases the reliability of effect inference. More importantly, examining the PH assumption allowed the detection of novel genetic variants with time‐varying associations. If validated in independent sets, these markers can help distinguish patients with different outcome risk during select time‐periods following diagnosis and therefore provide more specific prognostic estimates.

## 5 Conclusions

In conclusion, this study identified a novel common variant (which also showed an association in the TCGA patient dataset) and a number of rare variants, but no CNVs, that are associated with clinical outcomes in colorectal cancer. We also identified genetic variants with time‐varying associations, a traditionally understudied type of prognostic markers. Overall, identified variants/loci—if their prognostic values are validated in independent patient cohorts—can be used to stratify colorectal cancer patients into different risk groups and help guide the treatment strategies and clinic follow‐up in the future.

## Conflict of interest

The authors declare no conflict of interest.

## Author contributions

YY helped design the statistical approach, performed the imputations, conducted all statistical and bioinformatics analyses, interpreted the results, and drafted the manuscript. SW generated the INDEL/CNV data analyzed in this study. MC helped collect the outcome data. PP led the NFCCR. YEY conceptualized the study and led the statistical design. SS conceptualized, led, and helped design the study, helped collect patient‐related data, helped draft and revised the manuscript, and submitted the manuscript. All authors approved the final version of the manuscript.

### Peer Review

The peer review history for this article is available at https://publons.com/publon/10.1002/1878‐0261.13067.

## Supporting information


**Fig. S1**. Info scores of all imputed variants.
**Fig. S2**. Relationship between info score and MAF.
**Fig. S3**. Info scores of the imputed variants with MAF ≥ 0.05.
**Fig. S4**. Manhattan plot showing the SNP (i.e., rs7314075) with a p‐value that passed the 5×10^−06^ threshold (indicated by the red line) in the univariate Cox regression analysis (DSS; *dominant* genetic model).
**Fig. S5**. Manhattan plot showing the SNPs with their p‐values that passed the 5×10^−06^ threshold (indicated by the red line) in the univariate Cox regression analysis (DSS: *additive* genetic model).
**Fig. S6**. Regional plot of rs7314075 in univariate analysis (DSS; *dominant* genetic model).
**Fig. S7**. Regional plot of rs7314075 in univariate analysis (DSS; *additive* genetic model).
**Fig. S8**. QQ plot for the univariate DSS analysis under the dominant genetic model.
**Fig. S9**. QQ plot for the univariate DSS analysis under the additive genetic model.
**Fig. S10**. Plot of Eigenvalues of principal components (PCs).
**Fig. S11**. Expression levels of *ERP27* in colorectal tumors and normal tissues.
**Table S1**. CNVs/INDELs examined in this study.
**Table S2**. Pair‐wise Pearson correlation coefficients of clinico‐demographic variables, and MSI status in the SNP analysis cohort with 505 patients.
**Table S3**. The number of genetic variants analyzed in the univariate and multivariate analyses.
**Table S4**. Baseline characteristics of the TCGA colorectal cancer patient cohort.
**Table S5**. SNPs identified to be significantly associated with disease‐specific survival (DSS) in multivariate analysis under the *recessive* genetic model.
**Table S6**. SNPs identified to be significantly associated with recurrence/metastasis‐free survival (RMFS) in multivariate analysis under the *recessive* model.
**Table S7**. Top SNPs in multivariate analysis that have nominal/suggestive associations with recurrence/metastasis‐free survival (RMFS) under the dominant and additive genetic models.
**Table S8**. eQTLs (identified and high‐LD variants) in DSS and RMFS recessive models.
**Table S9**. Association between *WBP11* expression levels and consensus molecular subtypes (CMS).
**Table S10**. Top CNVs/INDELs in univariate analysis of the disease‐specific survival (DSS) and recurrence/metastasis‐free survival (RMFS).

## Data Availability

NFCCR data that support the findings of this study are available from the authors, Newfoundland Colorectal Cancer Registry/Memorial University, but restrictions apply to the availability of this data, and so data are not publicly available. The data from Newfoundland Colorectal Cancer Registry (NFCCR) used in this study cannot be made publicly available as patients were not consented to make their data publicly available or accessible. Imputation data are available from the authors and other data are available from the Newfoundland Colorectal Cancer Registry (NFCCR) upon reasonable request for researchers who meet the criteria for access to confidential data. Permission to obtain the data can be requested from authors (Yajun Yu; yy6084@mun.ca; imputation data only pending the other approvals), Newfoundland Colorectal Cancer Registry (Dr. Patrick Parfrey; pparfrey@mun.ca) and Research, Grant, and Contract Services (rgcs@mun.ca) at Memorial University of Newfoundland, St. John’s, NL, Canada, and the ethics approval shall be obtained from the Health Research Ethics Board (HREB), Ethics Office, Health Research Ethics Authority, Suite 200, 95 Bonaventure Avenue, St. John’s, NL, A1B 2X5, Canada. TCGA clinical data are publicly available in Genomic Data Commons (GDC) data portal (https://portal.gdc.cancer.gov), and to our knowledge, TCGA controlled germline genetic data only can be accessed after authorization is approved through Genotypes and Phenotypes (dbGaP) (https://dbgap.ncbi.nlm.nih.gov/aa/wga.cgi?page=login).
