# Supplementary material for: A comprehensive analysis of SNPs and CNVs identifies novel markers associated with disease outcomes in colorectal cancer
Source: Mol Oncol. 2021 Aug 5;15(12):3329–47. doi: 10.1002/1878-0261.13067 (PMC8637572; doi:10.1002/1878-0261.13067)
Supplement: Supplementary file 1 — Fig. S1. Info scores of all imputed variants. Fig. S2. Relationship between info score and MAF. Fig. S3. Info scores of the imputed variants with MAF ≥ 0.05. Fig. S4. Manhattan plot showing the SNP (i.e., rs7314075) with a p‐value that passed the 5×10^−06^ threshold (indicated by the red line) in the univariate Cox regression analysis (DSS; dominant genetic model). Fig. S5. Manhattan plot showing the SNPs with their p‐values that passed the 5×10^−06^ threshold (indicated by the red line) in the univariate Cox regression analysis (DSS: additive genetic model). Fig. S6. Regional plot of rs7314075 in univariate analysis (DSS; dominant genetic model). Fig. S7. Regional plot of rs7314075 in univariate analysis (DSS; additive genetic model). Fig. S8. QQ plot for the univariate DSS analysis under the dominant genetic model. Fig. S9. QQ plot for the univariate DSS analysis under the additive genetic model. Fig. S10. Plot of Eigenvalues of principal components (PCs). Fig. S11. Expression levels of ERP27 in colorectal tumors and normal tissues. Table S1. CNVs/INDELs examined in this study. Table S2. Pair‐wise Pearson correlation coefficients of clinico‐demographic variables, and MSI status in the SNP analysis cohort with 505 patients. Table S3. The number of genetic variants analyzed in the univariate and multivariate analyses. Table S4. Baseline characteristics of the TCGA colorectal cancer patient cohort. Table S5. SNPs identified to be significantly associated with disease‐specific survival (DSS) in multivariate analysis under the recessive genetic model. Table S6. SNPs identified to be significantly associated with recurrence/metastasis‐free survival (RMFS) in multivariate analysis under the recessive model. Table S7. Top SNPs in multivariate analysis that have nominal/suggestive associations with recurrence/metastasis‐free survival (RMFS) under the dominant and additive genetic models. Table S8. eQTLs (identified and high‐LD variants) in DSS and RMFS recessive models. Table [file MOL2-15-3329-s001.pdf]

Supporting materials for:

A comprehensive analysis of SNPs and CNVs identifies novel markers associated with disease outcomes in colorectal cancer

Yajun Yu[1,&], Salem Werdyani[1,&], Megan Carey[1], Patrick Parfrey[2], Yildiz E. Yilmaz[1,2,3], Sevtap Savas[1,4 *,&]

[1] Discipline of Genetics, Faculty of Medicine, Memorial University, St. John's, NL, Canada.

[2] Discipline of Medicine, Faculty of Medicine, Memorial University, St. John's, NL, Canada.

[3] Department of Mathematics and Statistics, Faculty of Science, Memorial University, St. John's, NL, Canada.

[4] Discipline of Oncology, Faculty of Medicine, Memorial University, St. John's, NL, Canada.

&Current affiliation: Division of Biomedical Sciences, Faculty of Medicine, Memorial University, St. John's, NL, Canada.

*Corresponding author:

Sevtap Savas, PhD, Division of Biomedical Sciences, Faculty of Medicine, Memorial University

300 Prince Philip Drive, Room M5M324, St. John`s, NL, Canada A1B 3V6

Email: savas@mun.ca

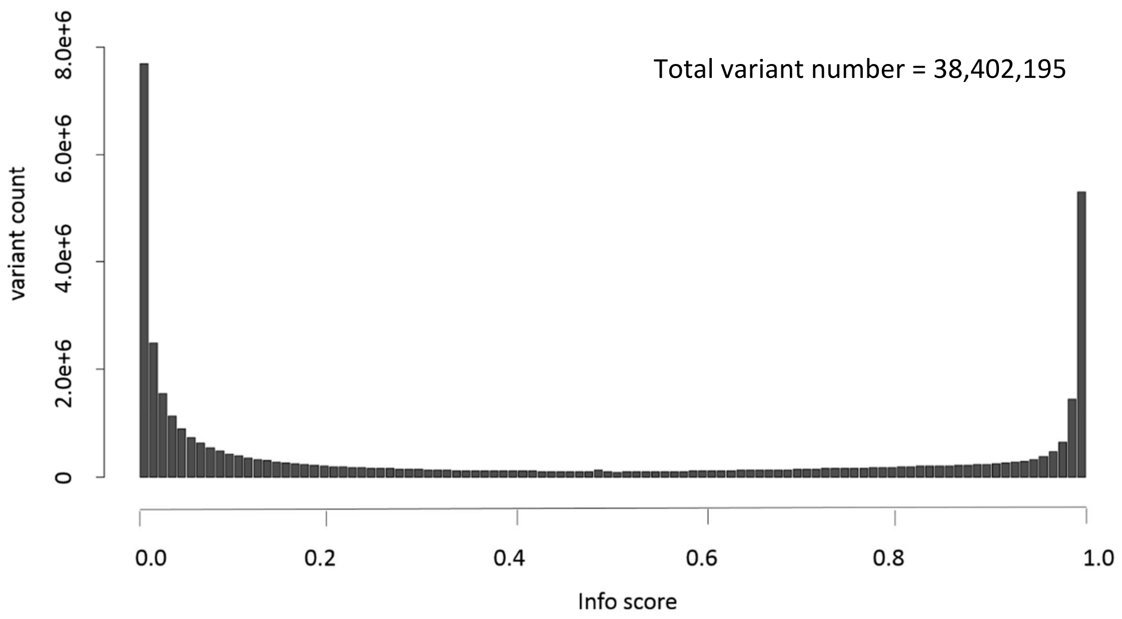

Supplementary Fig. 1. Info scores of all imputed variants. Variants were grouped by info score with 0.01 intervals. The majority of the variants had either very low or very high info scores.

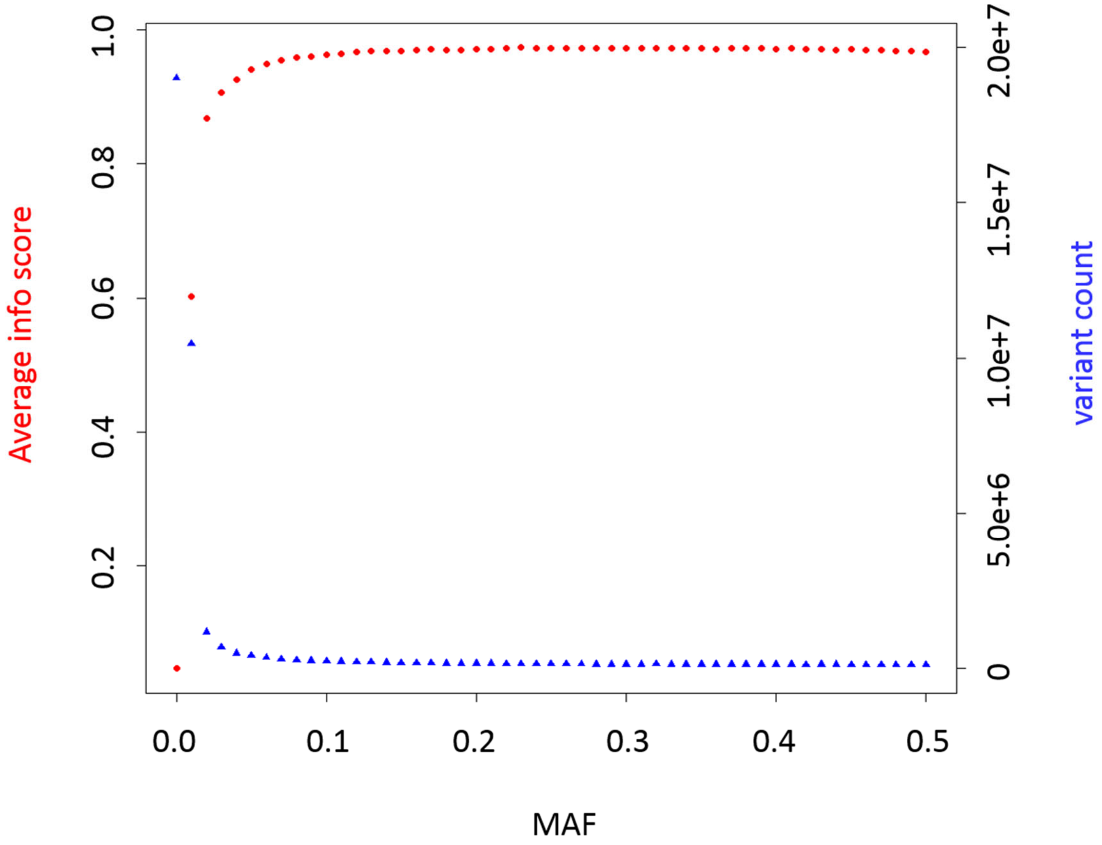

Supplementary Fig. 2. Relationship between info score and MAF. Imputed variants were grouped into 50 MAF-bins with each bin being defined as a 0.01 interval. The info scores of variants were averaged in each bin and marked with a red dot in the figure. The secondary axis indicates the number of variants in each MAF bin and the corresponding value is marked with blue triangles. The majority of the variants are with low MAFs (i.e. MAFs between 0 and 0.02) and were with low info scores (info score ≤ 0.7). Variants with higher MAFs showed high info scores, indicating that most of these variants were well-imputed SNPs.

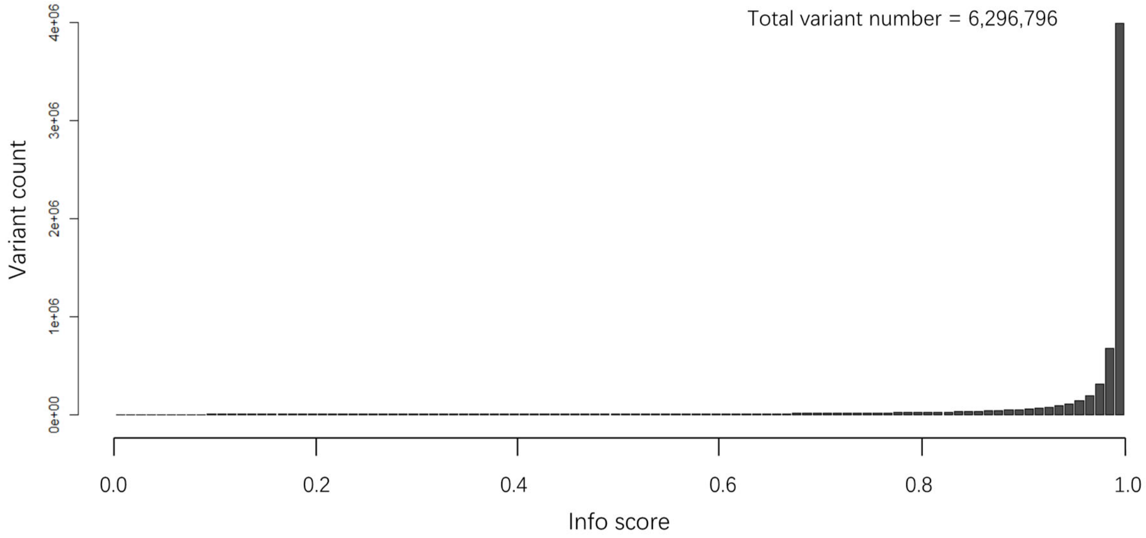

Supplementary Fig. 3. **Info scores of the imputed variants with MAF ≥ 0.05.** Variants were grouped by info score with 0.01 intervals. The majority of variants had very high info scores, indicating most of the imputed common variants were well-imputed variants.

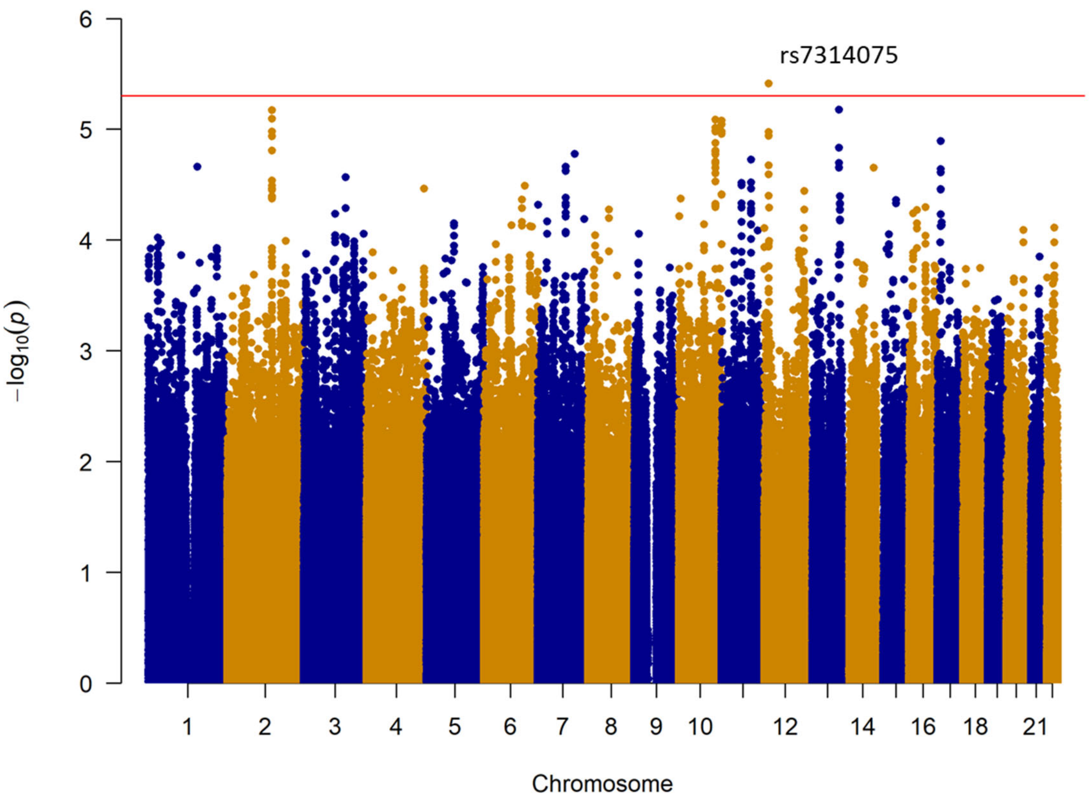

Supplementary Fig. 4. Manhattan plot showing the SNP (i.e. rs7314075) with a p value that passed the 5×10^{-06} threshold (indicated by the red line) in the univariate Cox regression analysis (DSS; *dominant* genetic model). Manhattan plot was generated using SNPs that satisfied the PH assumption in the univariate analysis. Note the SNP that is indicated in this figure is the SNP that passed the significance level of 5×10^{-08} in the multivariate analysis (see **Table 2**).

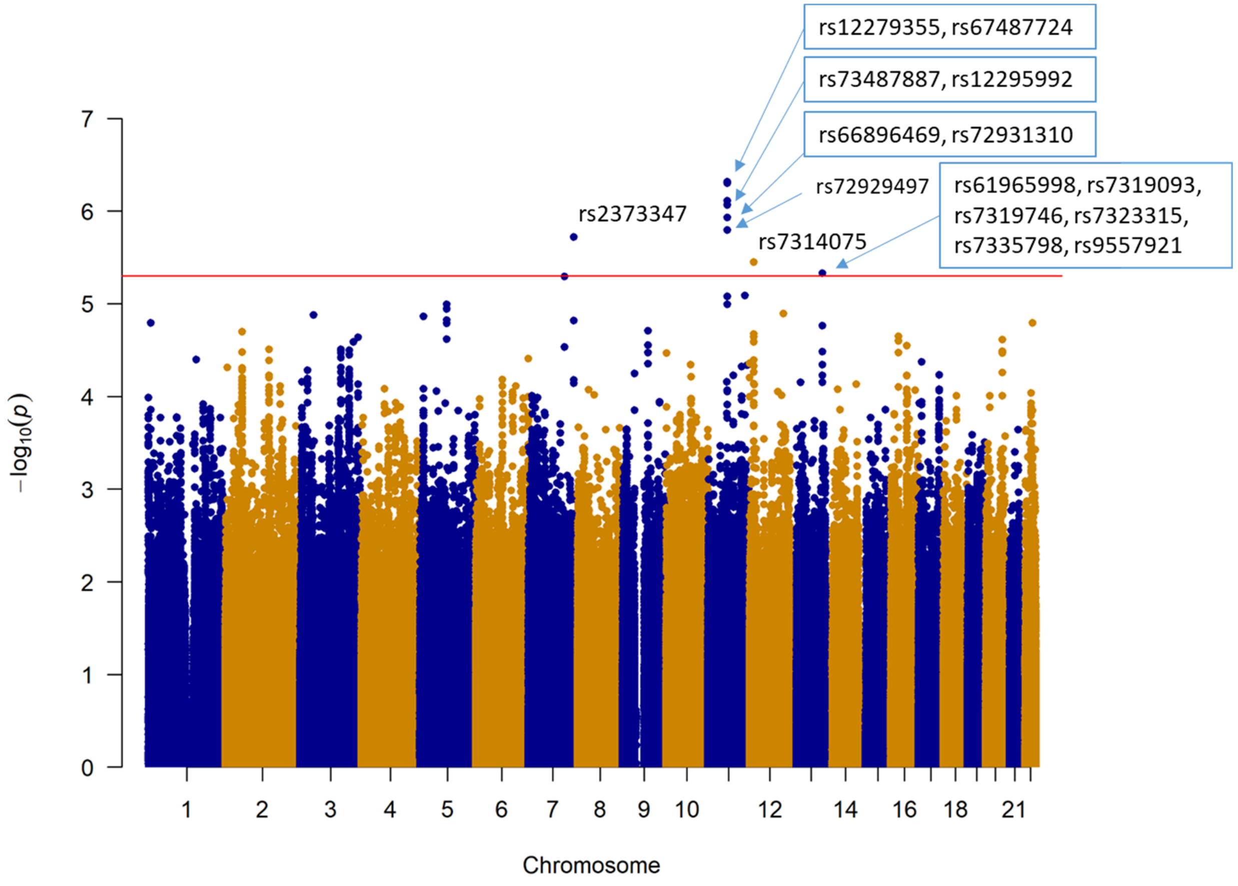

Supplementary Fig. 5. Manhattan plot showing the SNPs with their p values that passed the $5{\times}10^{-06}$ threshold (indicated by the red line) in the univariate Cox regression analysis (DSS: *additive* genetic model). Manhattan plot was generated using SNPs that satisfied the PH assumption in the univariate analysis. Note that rs7314075 is the SNP that passed the significance level of $5{\times}10^{-08}$ in the multivariate analysis (see **Table 2**).

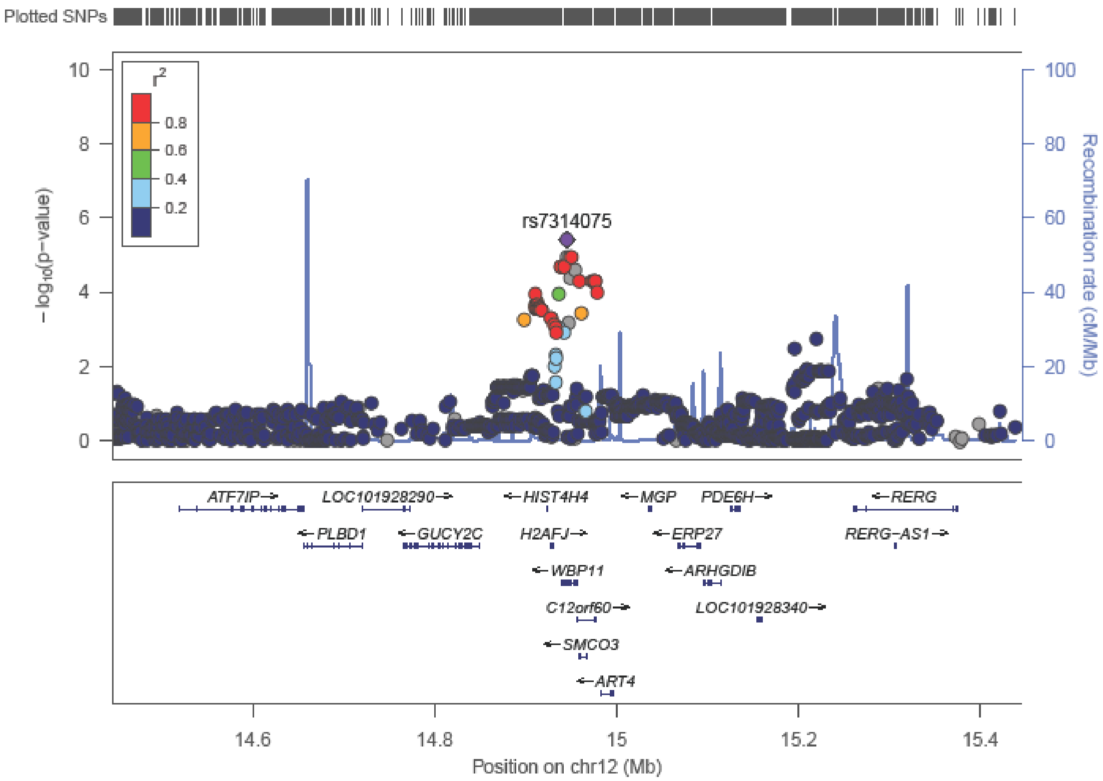

Supplementary Fig. 6. Regional plot of rs7314075 in univariate analysis (DSS; *dominant* genetic model).

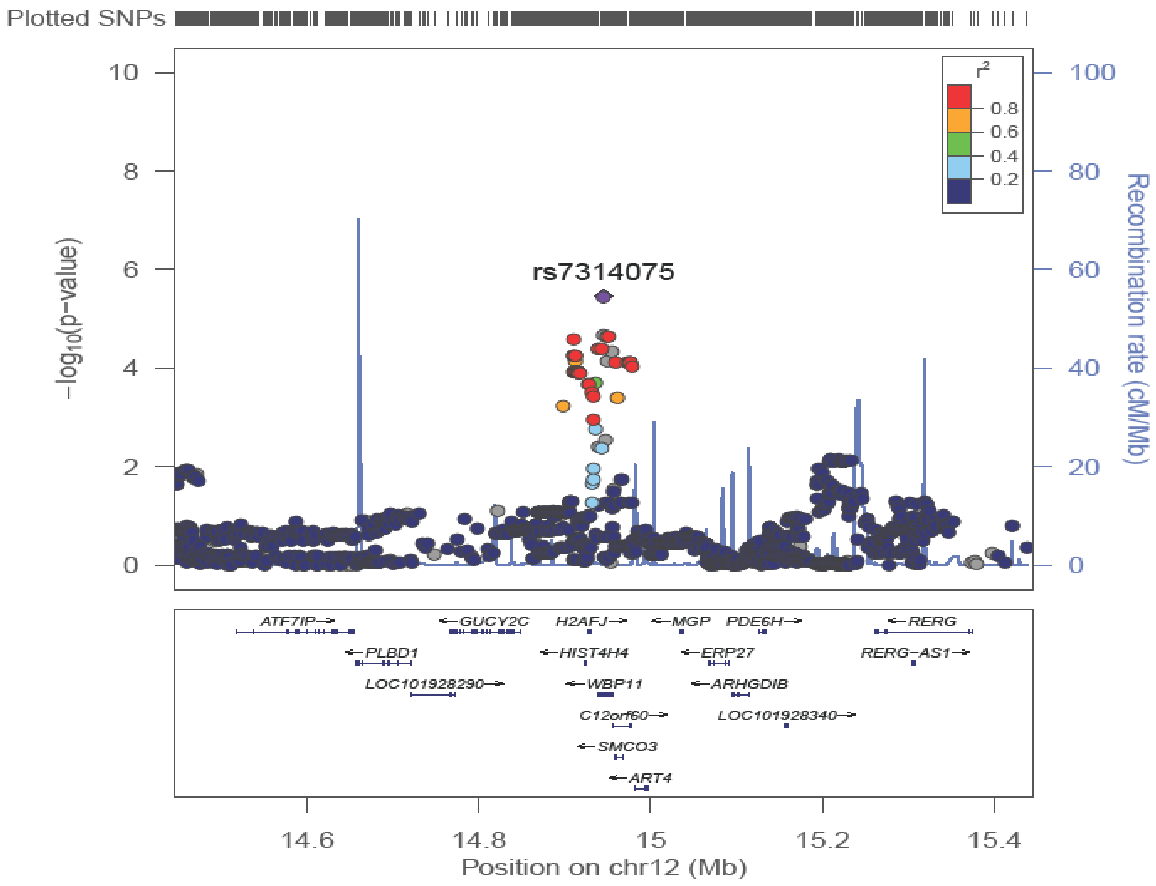

Supplementary Fig. 7. Regional plot of rs7314075 in univariate analysis (DSS; *additive* genetic model).

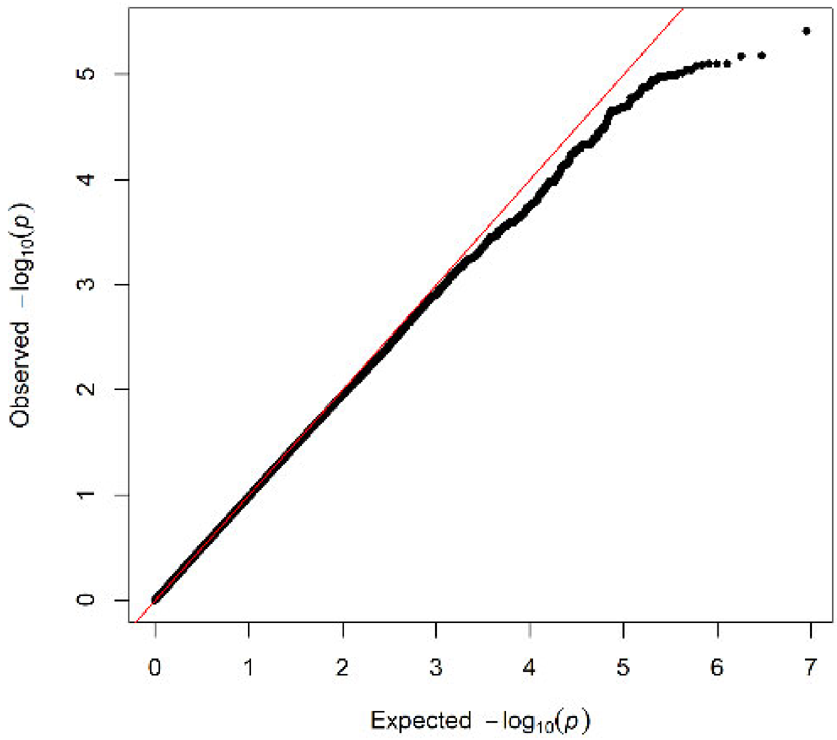

Supplementary Fig. 8. QQ plot for the univariate DSS analysis under the dominant genetic model. Plot was generated based on p-values of SNPs satisfied the PH assumption in the univariate analysis. Genomic inflation factor (λ) is 0.995.

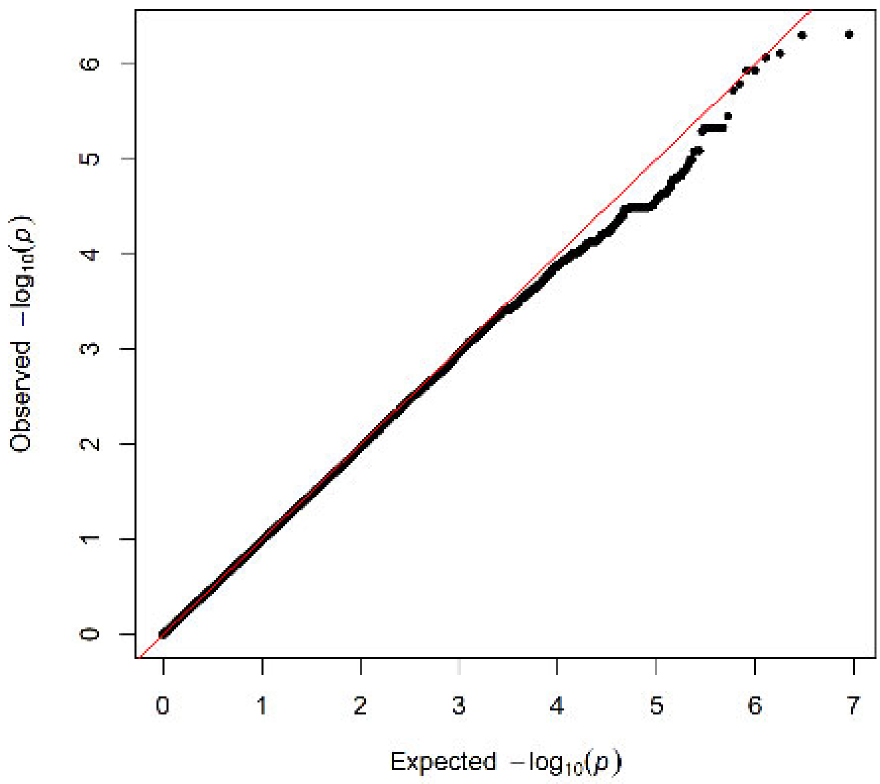

Supplementary Fig. 9. QQ plot for the univariate DSS analysis under the additive genetic model. Plot was generated based on p-values of SNPs satisfied the PH assumption in the univariate analysis. Genomic inflation factor (λ) is 0.987.

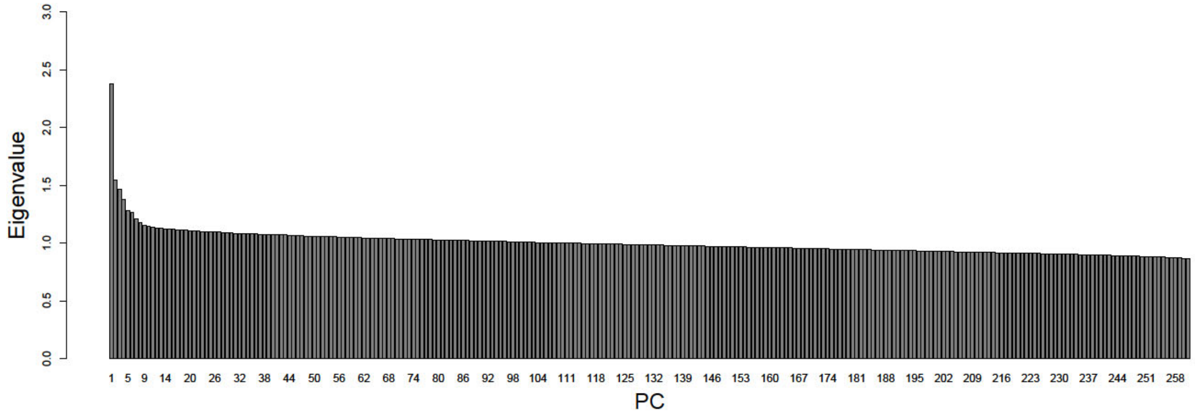

Supplementary Fig. 10. Plot of Eigenvalues of principal components (PCs).

i

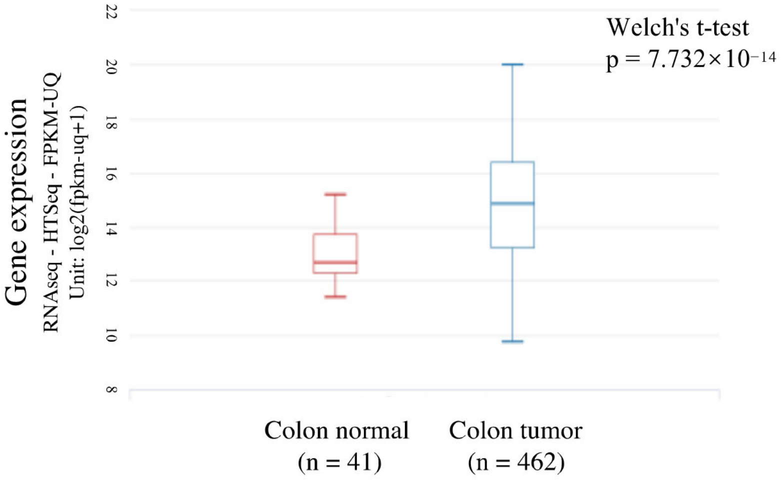

ii

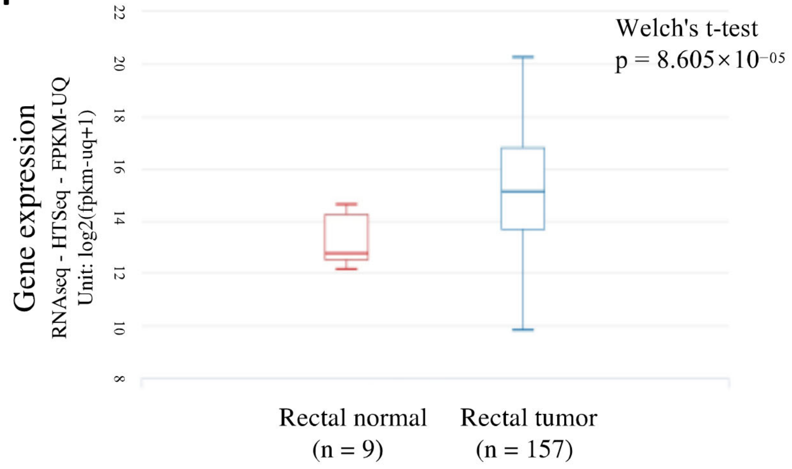

Supplementary Fig. 11. Expression levels of *ERP27* in colorectal tumors and normal tissues. Analysis was done in UCSC Xena [85] using the GDC TCGA COAD and READ data. In both datasets, primary tumors and adjacent normal tissues (noted as "solid tissue normal" in TCGA data) were selected (recurrent and metastatic tumors were excluded), and only the tumors and normal tissues with their anatomical sites noted as colon (in COAD) and rectum and rectosigmoid junction (in READ) were analyzed. i, gene expression in colon tumors and normal tissues from TCGA COAD cohort; ii, gene expression in rectal tumors and normal tissues from TCGA READ cohort. Expression of *ERP27* is significantly higher in colon and rectal tumors than in normal tissues. The number of patients in the colon and rectum tumor datasets is larger than those in the normal tissue datasets. This may explain why the gene expression levels in tumors have a higher variance compared to that in the normal tissues.

Supplementary Table 1. CNVs/INDELs examined in this study.

Variant	Variant type	Copy number status	0 copy frequency
Chr1:16152297-16153885	CNV	0, 2	0.10
Chr1:17676291-17677196	INDEL	0, 2	0.11
Chr1:58744143-58744663	INDEL	0, 2	0.76
Chr1:62082921-62083563	INDEL	0, 2	0.13
Chr1:72766413-72811692	CNV	0, 2	0.34
Chr1:80221868-80222895	CNV	0, 2	0.27
Chr1:89476427-89478432	CNV	0, 2	0.10
Chr1:92232111-92233227	CNV	0, 2	0.10
Chr1:106015878-106023356	CNV	0, 1, 2	0.14
Chr1:110187278-110188706	CNV	0, 2	0.34
Chr1:152556085-152586939	CNV	0, 2, 3	0.33
Chr1:158867802-158869733	CNV	0, 2	0.51
Chr1:159648791-159649527	INDEL	0, 2	0.56
Chr1:169207360-169241309	CNV	0, 1, 2	0.12
Chr1:179607382-179607936	INDEL	0, 2	0.13
Chr1:187717171-187722124	CNV	0, 1, 2	0.21
Chr1:194451546-194453078	CNV	0, 2	0.37
Chr1:207292578-207293178	INDEL	0, 2	0.45
Chr1:210078078-210085756	CNV	0, 2	0.37
Chr2:33224605-33227187	CNV	0, 2	0.10
Chr2:34523997-34524686	INDEL	0, 2	0.19
Chr2:34698447-34736476	CNV	0, 1, 2	0.15
Chr2:42346222-42347059	INDEL	0, 1, 2	0.19
Chr2:54565729-54567441	CNV	0, 2	0.32
Chr2:54565729-54567590	CNV	0, 1, 2	0.28
Chr2:56654397-56655541	CNV	0, 2	0.22
Chr2:70125092-70125504	INDEL	0, 2	0.15
Chr2:76773793-76775393	CNV	0, 2	0.55
Chr2:100103752-100105013	CNV	0, 2	0.20
Chr2:108855419-108856175	INDEL	0, 2	0.11
Chr2:126443281-126451762	CNV	0, 2	0.45
Chr2:127674739-127677079	CNV	0, 2	0.75
Chr2:146866008-146876881	CNV	0, 1, 2	0.21
Chr2:159959587-159961014	CNV	0, 2	0.31
Chr2:159959587-159961451	CNV	0, 2	0.18
Chr2:177268005-177271736	CNV	0, 2	0.61
Chr2:182856938-182857477	INDEL	0, 2	0.11
Chr2:194690106-194695458	CNV	0, 2	0.82

Chr2:215728845-215730688	CNV	0, 2	0.14
Chr2:227165698-227170955	CNV	0, 1, 2	0.11
Chr3:26450985-26452213	CNV	0, 2	0.22
Chr3:32102055-32106725	CNV	0, 1, 2	0.20
Chr3:47490712-47493338	CNV	0, 2	0.14
Chr3:68637537-68639440	CNV	0, 2	0.15
Chr3:68741426-68747798	CNV	0, 2	0.42
Chr3:80062959-80064447	CNV	0, 1, 2	0.37
Chr3:95465933-95468152	CNV	0, 2	0.11
Chr3:98411049-98414646	CNV	0, 2	0.15
Chr3:98900231-98902205	CNV	0, 2	0.56
Chr3:99628822-99629567	INDEL	0, 1, 2	0.10
Chr3:104278192-104279002	INDEL	0, 1, 2	0.11
Chr3:107038162-107040253	CNV	0, 2	0.10
Chr3:124936371-124936911	INDEL	0, 1, 2	0.17
Chr3:131708352-131713017	CNV	0, 1, 2	0.12
Chr3:136021052-136026101	CNV	0, 1, 2	0.13
Chr3:146387602-146390316	CNV	0, 2	0.47
Chr3:159257057-159257610	INDEL	0, 2	0.12
Chr3:162512207-162625930	CNV	0, 1, 2, 4	0.11
Chr3:162718988-162721962	CNV	0, 1, 2	0.18
Chr3:162765807-162769007	CNV	0, 2	0.52
Chr3:189737354-189740440	CNV	0, 1, 2	0.10
Chr3:192875738-192885153	CNV	0, 2, 4	0.48
Chr3:194398894-194400230	CNV	0, 2	0.36
Chr4:1602989-1603634	INDEL	0, 2	0.29
Chr4:6652161-6652800	INDEL	0, 1, 2	0.40
Chr4:6897543-6899625	CNV	0, 2	0.27
Chr4:10211268-10234260	CNV	0, 2, 3	0.49
Chr4:28421503-28422003	INDEL	0, 2	0.24
Chr4:46202844-46204937	CNV	0, 2	0.38
Chr4:61330156-61331760	CNV	0, 2	0.48
Chr4:61939506-61942200	CNV	0, 2	0.38
Chr4:64696875-64713573	CNV	0, 2	0.13
Chr4:91933043-91935779	CNV	0, 2	0.14
Chr4:115178984-115182290	CNV	0, 2, 4	0.17
Chr4:115928747-115929279	INDEL	0, 2	0.11
Chr4:133181351-133182077	INDEL	0, 2	0.13
Chr4:135433401-135435074	CNV	0, 2	0.27
Chr4:138966505-138967151	INDEL	0, 2	0.13
Chr4:142230896-142232849	CNV	0, 2	0.15

Chr4:146438871-146439991	CNV	0, 1, 2	0.16
Chr4:166003471-166004657	CNV	0, 2	0.78
Chr4:172374626-172378977	CNV	0, 2	0.11
Chr4:172989075-172992876	CNV	0, 2	0.22
Chr4:182056607-182057107	INDEL	0, 2	0.31
Chr4:186441932-186444023	CNV	0, 2	0.56
Chr4:186441932-186444110	CNV	0, 2	0.20
Chr4:187093557-187098071	CNV	0, 2	0.31
Chr5:1178511-1180425	CNV	0, 2	0.18
Chr5:1924651-1925051	INDEL	0, 2	0.68
Chr5:10273607-10274711	CNV	0, 2	0.52
Chr5:12811506-12819198	CNV	0, 1, 2	0.20
Chr5:19375544-19376421	INDEL	0, 1, 2	0.14
Chr5:21450792-21452439	CNV	0, 2	0.14
Chr5:57323612-57333211	CNV	0, 2	0.63
Chr5:60001832-60003352	CNV	0, 2	0.34
Chr5:83947987-83954795	CNV	0, 2	0.73
Chr5:83948595-83954795	CNV	0, 2	0.24
Chr5:90500630-90502051	CNV	0, 2	0.56
Chr5:97401582-97402715	CNV	0, 2	0.17
Chr5:98345182-98347056	CNV	0, 2	0.46
Chr5:106324802-106326299	CNV	0, 2	0.18
Chr5:119380452-119383902	CNV	0, 2	0.29
Chr5:135115551-135120517	CNV	0, 1, 2	0.24
Chr5:147553186-147554186	CNV	0, 2	0.33
Chr6:666535-667756	CNV	0, 2	0.39
Chr6:18402172-18402672	INDEL	0, 2	0.40
Chr6:32455482-32484368	CNV	0, 2	0.15
Chr6:32778882-32779506	INDEL	0, 2	0.20
Chr6:51736175-51736742	INDEL	0, 1, 2	0.25
Chr6:53929777-53933874	CNV	0, 2	0.47
Chr6:65347533-65349159	CNV	0, 2	0.29
Chr6:74592225-74599512	CNV	0, 1, 2	0.13
Chr6:77097876-77100253	CNV	0, 2	0.20
Chr6:77097876-77100461	CNV	0, 2	0.21
Chr6:86624320-86625771	CNV	0, 2	0.10
Chr6:89921782-89922171	INDEL	0, 2	0.32
Chr6:95193423-95194280	INDEL	0, 1, 2	0.35
Chr6:100034580-100035230	INDEL	0, 2	0.64
Chr6:114224070-114224975	INDEL	0, 2	0.11
Chr6:134269008-134269657	INDEL	0, 2	0.33

Chr6:141549108-141549906	INDEL	0, 2	0.13
Chr6:165724739-165731496	CNV	0, 2	0.21
Chr6:167488211-167489090	INDEL	0, 2	0.18
Chr7:22434643-22436658	CNV	0, 2	0.15
Chr7:24038309-24039976	CNV	0, 2	0.42
Chr7:31315876-31318783	CNV	0, 1, 2	0.23
Chr7:51594426-51598253	CNV	0, 2, 3	0.18
Chr7:62366067-62369218	CNV	0, 2	0.12
Chr7:70421545-70425749	CNV	0, 2	0.39
Chr7:73829165-73831200	CNV	0, 2	0.30
Chr7:89810608-89811996	CNV	0, 2	0.13
Chr7:89810608-89812114	CNV	0, 2	0.14
Chr7:93541865-93542465	INDEL	0, 2	0.36
Chr7:110182015-110188407	CNV	0, 1, 2	0.10
Chr7:118831427-118834466	CNV	0, 2	0.22
Chr7:126048572-126051369	CNV	0, 2	0.12
Chr7:126048572-126051476	CNV	0, 2	0.18
Chr7:133785061-133797965	CNV	0, 1, 2	0.11
Chr7:148074379-148076266	CNV	0, 2	0.67
Chr8:594761-599201	CNV	0, 2	0.68
Chr8:4122961-4124156	CNV	0, 2	0.43
Chr8:11245641-11247049	CNV	0, 2	0.18
Chr8:25066884-25070636	CNV	0, 2	0.60
Chr8:39233344-39387179	CNV	0, 1, 2	0.17
Chr8:40774744-40779338	CNV	0, 2	0.30
Chr8:42191238-42193395	CNV	0, 2	0.14
Chr8:42191238-42193599	CNV	0, 2	0.59
Chr8:75364528-75366830	CNV	0, 2	0.35
Chr8:112294125-112296209	CNV	0, 1, 2	0.38
Chr8:127192269-127194257	CNV	0, 2	0.10
Chr8:137160319-137163816	CNV	0, 2	0.26
Chr8:138742985-138743769	INDEL	0, 2	0.76
Chr9:17910043-17911627	CNV	0, 2	0.64
Chr9:22496202-22502596	CNV	0, 1, 2	0.15
Chr9:23362799-23377416	CNV	0, 1, 2	0.18
Chr9:31291381-31292431	CNV	0, 2	0.13
Chr9:71741217-71743100	CNV	0, 2	0.53
Chr9:71895369-71896250	INDEL	0, 2	0.55
Chr9:89154979-89155745	INDEL	0, 2	0.57
Chr9:101309058-101311079	CNV	0, 2	0.24
Chr9:131412549-131413853	CNV	0, 2	0.32

Chr9:131412549-131413885	CNV	0, 2	0.49
Chr9:136625265-136626037	INDEL	0, 2	0.23
Chr9:138214337-138217541	CNV	0, 2	0.16
Chr9:138479177-138480145	INDEL	0, 2	0.36
Chr10:4290129-4291584	CNV	0, 2	0.82
Chr10:4708627-4710298	CNV	0, 2	0.53
Chr10:7077551-7078246	INDEL	0, 1, 2	0.19
Chr10:27000558-27001814	CNV	0, 2	0.35
Chr10:31443722-31444976	CNV	0, 1, 2	0.22
Chr10:67306995-67314427	CNV	0, 1, 2	0.10
Chr10:78255873-78260694	CNV	0, 2	0.65
Chr10:89275888-89276407	INDEL	0, 1, 2	0.43
Chr10:93633430-93634441	CNV	0, 2	0.22
Chr10:95545536-95546273	INDEL	0, 2	0.89
Chr10:107950711-107951550	INDEL	0, 2	0.70
Chr10:114113589-114116575	CNV	0, 2	0.13
Chr10:122226947-122228534	CNV	0, 2	0.15
Chr11:5760106-5762286	CNV	0, 2	0.14
Chr11:9324025-9324496	INDEL	0, 2	0.23
Chr11:29967596-29968238	INDEL	0, 2	0.13
Chr11:31394060-31397428	CNV	0, 1, 2	0.13
Chr11:45430401-45431405	CNV	0, 2	0.30
Chr11:66712229-66713105	INDEL	0, 2	0.23
Chr11:93021163-93022144	INDEL	0, 2	0.19
Chr11:104267791-104272611	CNV	0, 2	0.49
Chr12:12026506-12026937	INDEL	0, 2	0.12
Chr12:16420184-16420943	INDEL	0, 2	0.18
Chr12:30478334-30480626	CNV	0, 1, 2	0.15
Chr12:45903118-45909531	CNV	0, 1, 2	0.29
Chr12:48709423-48710624	CNV	0, 1, 2	0.10
Chr12:60522630-60524927	CNV	0, 1, 2	0.26
Chr12:90488020-90491702	CNV	0, 2	0.18
Chr13:27050284-27052028	CNV	0, 2	0.19
Chr13:39057351-39060049	CNV	0, 2	0.43
Chr13:39934551-39935151	INDEL	0, 2	0.37
Chr13:49533568-49536464	CNV	0, 1, 2	0.12
Chr13:51069352-51072600	CNV	0, 2	0.54
Chr13:72478244-72480589	CNV	0, 1, 2	0.33
Chr13:72845850-72846775	INDEL	0, 1, 2	0.47
Chr13:90862850-90864719	CNV	0, 2	0.25
Chr13:99254450-99257146	CNV	0, 1, 2	0.10

Chr13:101894125-101896318	CNV	0, 2	0.31
Chr13:106449351-106449814	INDEL	0, 2	0.14
Chr14:20551808-20552611	INDEL	0, 2	0.29
Chr14:40615179-40617594	CNV	0, 2	0.23
Chr14:82499370-82503183	CNV	0, 2	0.38
Chr15:39372623-39373245	INDEL	0, 2	0.17
Chr15:71881673-71882625	INDEL	0, 2	0.10
Chr15:76891342-76895185	CNV	0, 1, 2	0.25
Chr15:77330786-77332606	CNV	0, 2	0.43
Chr15:91981864-91983360	CNV	0, 2	0.28
Chr16:23048233-23049446	CNV	0, 2	0.40
Chr16:48509311-48510755	CNV	0, 1, 2	0.26
Chr16:57326658-57327126	INDEL	0, 2	0.11
Chr16:58647399-58649650	CNV	0, 2	0.23
Chr16:76540062-76543447	CNV	0, 2	0.50
Chr16:78373700-78384735	CNV	0, 1, 2	0.24
Chr17:724239-724598	INDEL	0, 2	0.35
Chr17:14190726-14191429	INDEL	0, 2	0.29
Chr17:35755867-35758648	CNV	0, 1, 2	0.15
Chr17:41517334-41518185	INDEL	0, 2	0.11
Chr17:51855247-51859534	CNV	0, 1, 2	0.13
Chr17:55688120-55689796	CNV	0, 2	0.36
Chr17:76282555-76282929	INDEL	0, 2	0.28
Chr18:5324676-5326221	CNV	0, 2	0.27
Chr18:24571673-24572190	INDEL	0, 2	0.17
Chr18:35306101-35306609	INDEL	0, 2	0.49
Chr18:38862147-38868004	CNV	0, 2	0.60
Chr18:38864903-38868004	CNV	0, 2	0.23
Chr18:47695103-47698268	CNV	0, 2	0.11
Chr18:54946766-54948517	CNV	0, 2	0.56
Chr18:63766950-63769066	CNV	0, 2	0.11
Chr18:75267039-75267968	INDEL	0, 2	0.33
Chr18:77310162-77312078	CNV	0, 1, 2	0.42
Chr19:2909643-2910369	INDEL	0, 2	0.79
Chr19:5510301-5510667	INDEL	0, 2	0.11
Chr19:12694963-12697389	CNV	0, 2	0.48
Chr19:13776129-13776658	INDEL	0, 2	0.12
Chr19:15046722-15047605	INDEL	0, 2	0.28
Chr19:31287833-31289043	CNV	0, 2	0.30
Chr19:51406972-51407935	INDEL	0, 2	0.11
Chr20:1389773-1390682	INDEL	0, 2	0.12

Chr20:1389773-1391436	CNV	0, 2	0.14
Chr21:16588414-16589135	INDEL	0, 2	0.19
Chr21:19327135-19328810	CNV	0, 1, 2	0.11
Chr21:44970373-44973184	CNV	0, 2	0.30
Chr21:47388151-47389593	CNV	0, 2	0.28
Chr22:18058001-18059664	CNV	0, 2	0.80
Chr22:24274775-24276797	CNV	0, 2	0.17
Chr22:24365041-24367511	CNV	0, 2	0.14
Chr22:35645524-35646052	INDEL	0, 2	0.43
Chr22:37143405-37146870	CNV	0, 2	0.50
Chr22:39295546-39298533	CNV	0, 1, 2	0.18

CNV, copy number variation; INDEL, insertion/deletion. Data based on a previous study [29] of our group.

Supplementary Table 2. Pair-wise Pearson correlation coefficients of clinico-demographic variables, and MSI status in the SNP analysis cohort with 505 patients.

	Sex	Grade	Histology	Location	Stage	MSI status	Adjuvant chemotherapy treatment	Adjuvant radiotherapy treatment
Sex	1.00	0.02	-0.09	0.09	0.04	-0.12	0.08	0.06
Grade		1.00	0.07	0.01	0.08	0.13	0.05	0.05
Histology			1.00	-0.12	0.09	0.09	0.03	-0.09
Location				1.00	-0.04	-0.19	0.22	0.69
Stage					1.00	-0.09	0.40	0.14
MSI status						1.00	-0.04	-0.11
Adjuvant chemotherapy treatment							1.00	0.51
Adjuvant radiotherapy treatment								1.00

MSI, microsatellite instability.

Supplementary Table 3. The number of genetic variants analyzed in the univariate and multivariate analyses.

SNPs						
	**Additive model**		**Dominant model**		**Recessive model**	
	Number of variants	*Number of variants entered into the multivariate analysis	Number of variants	*Number of variants entered into the multivariate analysis	Number of variants	*Number of variants entered into the multivariate analysis
DSS - univariate						
Satisfying the PH assumption (No cut-off time)	4,464,856	15	4,470,070	1	4,507,677	271
Satisfying the PH assumption using 5 years as the cut-off time	244,790	10	239,574	2	203,103	149
**Still violating PH assumption after using the 5 years as the cut-off time	1,663	NA	1,665	NA	529	NA
RMFS - univariate						
Satisfying the PH assumption (No cut-off time)	4,471,130	152	4,470,972	3	4,497,910	338
Satisfying the PH assumption using 5 years as the cut-off time	179,755	17	181,300	0	169,358	315
**Still violating the PH assumption after using 5 years as the cut-off time	60,424	NA	59,037	NA	44,041	NA

CNVs/INDELs		
	Number of variants	*Number of variants entered into the multivariate analysis
DSS - univariate		
Satisfying the PH assumption (No cut-off time)	235	0
Satisfying the PH assumption using 5 years as the cut-off time	19	0
**Still violating the PH assumption after using 5 years as the cut-off time	0	NA
RMFS - univariate		
Satisfying the PH assumption (No cut-off time)	243	0
Satisfying the PH assumption using 5 years as the cut-off time	10	0
**Still violating the PH assumption after using 5 years as the cut-off time	1	NA

CI, confidence interval; CNV, copy number variation; DSS, disease-specific survival; INDEL, insertion/deletion; NA, not applicable; PH, proportional hazards; RMFS, recurrence/metastasis-free survival; SNP, single nucleotide polymorphism.
*, the univariate p value threshold for variants to enter into the multivariate analysis is 5×10^{-06}. **, excluded from further analysis. Note that SNPs that passed the univariate p value threshold were not entered into multivariate analysis if their upper limits of the 95% CIs were infinity.

Supplementary Table 4. Baseline characteristics of the TCGA colorectal cancer patient cohort.

Variable	Number (n=262 in total)	%
**Tumor location**		
Colon	188	71.76
Rectum	74	28.24
**Stage**		
I	41	15.65
II	91	34.73
III	82	31.30
IV	38	14.50
Unknown	10	3.82
**MSI**		
MSI-L/MSS	217	82.82
MSI-H	44	16.79
Unknown	1	0.38
**Follow-up time**		
Median (range)	2.04 (0 - 11.70)	-
**DSS status**		
Death from other causes or alive	218	83.21
Death from colorectal cancer	24	9.16
Unknown	20	7.63

DSS, disease-free survival; MSI, microsatellite instability; MSI-H, microsatellite instability-high; MSI-L, microsatellite instability-low; MSS, microsatellite stable. Data based on the GDC (https://portal.gdc.cancer.gov/) and a study [78] published in 2018.

Supplementary Table 5. SNPs identified to be significantly associated with disease-specific survival (DSS) in multivariate analysis under the *recessive* genetic model.

Variant	Chr	Position	Minor/ major allele	MAF	Variant type	Info Score	Time period post-diagnosis	#HR (95% CI)	p value	p value of the PH assump-tion test	*Located region
rs28552674	1	235583778	T/C	0.10	Imputed	0.998	-	44.16 (12.04, 161.98)	**$1.12{\times}10^{-08}$**	0.89	Intron of *TBCE*
rs12758637	1	235584193	A/G	0.10	Imputed	1	-	44.16 (12.04, 161.98)	**$1.12{\times}10^{-08}$**	0.89	Intron of *TBCE*
rs11579933	1	235588409	A/C	0.10	Imputed	1	-	44.16 (12.04, 161.98)	**$1.12{\times}10^{-08}$**	0.89	Intron of *TBCE*
kgp2690683	1	235590559	A/G	0.10	Genotyped	-	-	44.16 (12.04, 161.98)	**$1.12{\times}10^{-08}$**	0.89	Intron of *TBCE*
rs71640701	1	235608749	A/G	0.10	Imputed	0.998	-	44.16 (12.04, 161.98)	**$1.12{\times}10^{-08}$**	0.89	Intron of *TBCE*
rs72239609	1	235609498	CT/C	0.10	Imputed	0.983	-	45.89 (12.50, 168.44)	**$8.03{\times}10^{-09}$**	0.85	Intron of *TBCE*
rs6429094	1	235611093	G/A	0.11	Genotyped	-	-	44.16 (12.04, 161.98)	**$1.12{\times}10^{-08}$**	0.89	Intron of *TBCE,* 3' UTR of *B3GALNT2*
rs35242859	1	235612394	CAGT T/C	0.10	Imputed	1	-	44.16 (12.04, 161.98)	**$1.12{\times}10^{-08}$**	0.89	3' UTR of *B3GALNT2*
rs358373	3	13188055	G/A	0.12	Imputed	0.970	-	17.87 (6.45, 49.49)	**$2.88{\times}10^{-08}$**	0.44	5' of *IQSEC1*
rs530425	3	13189919	A/G	0.12	Imputed	0.986	-	17.36 (6.31, 47.76)	**$3.28{\times}10^{-08}$**	0.59	5' of *IQSEC1*
rs140970549	3	164282763	T/TTT C	0.17	Imputed	1	-	9.35 (4.33, 20.22)	**$1.31{\times}10^{-08}$**	0.93	3' of *SI*
rs58844954	3	164330365	C/T	0.17	Imputed	0.995	-	9.32 (4.31, 20.15)	**$1.38{\times}10^{-08}$**	0.93	3' of *SI*

Chr, chromosome; CI, confidence interval; HR, hazard ratio; MAF, minor allele frequency; PH, proportional hazards. #, Hazard ratio was estimated under the recessive genetic model for AA vs [AB+BB], where A is the minor allele and B is the major allele. *, Gene annotation is obtained from the UCSC database ("UCSC genes" from the UCSC browser [GRCh37/hg19]) [96], and only the overlapped (for SNPs within genes) or the closest (for SNPs in Intergenic regions) genes are shown in this Table. 3', downstream of the gene. 5', upstream of the gene. Models are adjusted for MSI status,

disease stage, tumor location (6 years as the cut-off time point), adjuvant chemotherapy and radiotherapy statuses (7 years as the cut-off time point for adjuvant radiotherapy). SNPs that are in high-LD ($r^2 > 0.8$) with each other on the same chromosome are highlighted.

Supplementary Table 6. SNPs identified to be significantly associated with recurrence/metastasis-free survival (RMFS) in multivariate analysis under the *recessive* model.

Variant	Chr	Position	Minor/ major allele	MAF	Variant type	Info score	Time period post-diagno-sis	#HR (95% CI)	p value	p value of the PH assump-tion test	*Located region
rs4534237	2	114160484	G/C	0.12	Imputed	0.975	-	9.69 (4.50, 20.85)	**6.27×10^{-09}**	0.49	5' of *CBWD2*
rs75261537	2	114162255	C/A	0.12	Imputed	0.975	-	9.69 (4.50, 20.85)	**6.27×10^{-09}**	0.49	5' of *CBWD2*
rs72641537	4	64327395	T/C	0.10	Imputed	0.982	-	20.18 (6.91, 58.9)	**3.86×10^{-08}**	0.77	3' of *TECRL*
rs11307057	4	94764199	T/TG	0.16	Imputed	0.98	-	8.29 (3.91, 17.58)	**3.49×10^{-08}**	0.52	3’ of *ATOH1*
rs11193950	10	109966710	T/C	0.14	Imputed	0.993	-	20.54 (8.47, 49.82)	**2.33×10^{-11}**	0.54	5' of *SORCS1*
rs10884600	10	109969396	G/A	0.14	Genotyp ed	-	-	20.85 (8.59, 50.56)	**1.84×10^{-11}**	0.54	5' of *SORCS1*
rs11193953	10	109971822	C/T	0.14	Imputed	1	-	20.85 (8.59, 50.56)	**1.84×10^{-11}**	0.54	5' of *SORCS1*
rs11193956	10	109972622	A/G	0.14	Imputed	1	-	20.85 (8.59, 50.56)	**1.84×10^{-11}**	0.54	5' of *SORCS1*
rs144602401	10	109973954	C/CTA TT	0.14	Imputed	1	-	20.85 (8.59, 50.56)	**1.84×10^{-11}**	0.54	5' of *SORCS1*
rs11193958	10	109974184	T/C	0.15	Imputed	0.973	-	17.08 (7.49, 38.96)	**1.52×10^{-11}**	0.45	5' of *SORCS1*
rs113965753	10	109975195	A/AG	0.14	Imputed	1	-	20.85 (8.59, 50.56)	**1.84×10^{-11}**	0.54	5' of *SORCS1*
rs11193959	10	109979273	C/T	0.14	Imputed	1	-	20.85 (8.59, 50.56)	**1.84×10^{-11}**	0.54	5' of *SORCS1*
rs11193962	10	109983961	A/G	0.14	Imputed	1	-	20.85 (8.59, 50.56)	**1.84×10^{-11}**	0.54	5' of *SORCS1*
rs12266409	10	109986116	G/A	0.14	Imputed	1	-	20.85 (8.59, 50.56)	**1.84×10^{-11}**	0.54	5' of *SORCS1*
rs12357671	10	109987906	T/C	0.14	Imputed	1	-	20.85 (8.59, 50.56)	**1.84×10^{-11}**	0.54	5' of *SORCS1*

rs10509868	10	109988645	G/A	0.14	Genotyp ed	-	-	20.85 (8.59, 50.56)	**$1.84{\times}10^{-11}$**	0.54	5' of *SORCS1*
rs12355000	10	109988766	C/A	0.14	Imputed	1	-	20.85 (8.59, 50.56)	**$1.84{\times}10^{-11}$**	0.54	5' of *SORCS1*
rs11193967	10	109996302	C/T	0.14	Imputed	1	-	20.85 (8.59, 50.56)	**$1.84{\times}10^{-11}$**	0.54	5' of *SORCS1*
rs11193968	10	109996688	T/A	0.14	Imputed	1	-	20.85 (8.59, 50.56)	**$1.84{\times}10^{-11}$**	0.54	5' of *SORCS1*
rs11193971	10	109998171	A/T	0.14	Imputed	1	-	20.85 (8.59, 50.56)	**$1.84{\times}10^{-11}$**	0.54	5' of *SORCS1*
rs11193975	10	109999737	A/G	0.14	Imputed	1	-	20.85 (8.59, 50.56)	**$1.84{\times}10^{-11}$**	0.54	5' of *SORCS1*
rs11193976	10	110003244	T/C	0.14	Imputed	1	-	20.85 (8.59, 50.56)	**$1.84{\times}10^{-11}$**	0.54	5' of *SORCS1*
rs7920125	10	110003692	T/C	0.14	Imputed	1	-	20.85 (8.59, 50.56)	**$1.84{\times}10^{-11}$**	0.54	5' of *SORCS1*
rs7920134	10	110003720	G/C	0.14	Imputed	1	-	20.85 (8.59, 50.56)	**$1.84{\times}10^{-11}$**	0.54	5' of *SORCS1*
rs11193977	10	110004373	A/C	0.14	Imputed	1	-	20.85 (8.59, 50.56)	**$1.84{\times}10^{-11}$**	0.54	5' of *SORCS1*
rs11193978	10	110004533	A/G	0.14	Imputed	1	-	20.85 (8.59, 50.56)	**$1.84{\times}10^{-11}$**	0.54	5' of *SORCS1*
rs58143932	10	110005552	T/C	0.14	Imputed	1	-	20.85 (8.59, 50.56)	**$1.84{\times}10^{-11}$**	0.54	5' of *SORCS1*
rs11193980	10	110006462	A/C	0.14	Imputed	1	-	20.85 (8.59, 50.56)	**$1.84{\times}10^{-11}$**	0.54	5' of *SORCS1*
rs112377485	10	110016099	G/GA	0.15	Imputed	0.991	-	20.6 (8.49, 49.99)	**$2.23{\times}10^{-11}$**	0.55	5' of *SORCS1*
rs17124169	10	110017139	C/A	0.14	Imputed	1	-	20.85 (8.59, 50.56)	**$1.84{\times}10^{-11}$**	0.54	5' of *SORCS1*
rs11193989	10	110021384	T/C	0.14	Imputed	1	-	20.85 (8.59, 50.56)	**$1.84{\times}10^{-11}$**	0.54	5' of *SORCS1*
rs11193990	10	110021388	T/C	0.14	Imputed	1	-	20.85 (8.59, 50.56)	**$1.84{\times}10^{-11}$**	0.54	5' of *SORCS1*
rs9988666	10	110032277	C/T	0.14	Imputed	0.996	-	20.72 (8.54, 50.25)	**$1.99{\times}10^{-11}$**	0.55	5' of *SORCS1*
rs10884603	10	110033909	A/C	0.14	Imputed	0.995	-	20.72 (8.54, 50.25)	**$1.99{\times}10^{-11}$**	0.55	5' of *SORCS1*

rs10884604	10	110035202	G/A	0.14	Imputed	0.994	-	20.72 (8.54, 50.25)	**1.99×10^{-11}**	0.55	5' of *SORCS1*
rs10884605	10	110035573	A/T	0.14	Imputed	0.994	-	20.72 (8.54, 50.25)	**1.99×10^{-11}**	0.55	5' of *SORCS1*
rs10884606	10	110037946	A/C	0.14	Imputed	0.993	-	20.72 (8.54, 50.25)	**1.99×10^{-11}**	0.55	5' of *SORCS1*
rs117213049	10	110045564	T/A	0.14	Imputed	0.972	-	21.24 (8.74, 51.63)	**1.54×10^{-11}**	0.57	5' of *SORCS1*
rs77206409	11	19420899	A/C	0.13	Imputed	0.991	-	34.91 (13.11, 92.97)	**1.17×10^{-12}**	0.79	Intron of *NAV2*
rs79632817	11	19426141	C/G	0.12	Imputed	0.972	-	35.59 (13.32, 95.15)	**1.08×10^{-12}**	0.80	Intron of *NAV2*
rs78348500	11	19427476	G/T	0.12	Imputed	0.967	-	35.59 (13.32, 95.15)	**1.08×10^{-12}**	0.80	Intron of *NAV2*
rs76656890	11	19427968	T/G	0.12	Imputed	0.963	-	35.55 (13.30, 95.03)	**1.09×10^{-12}**	0.80	Intron of *NAV2*
rs12225106	11	86443409	A/G	0.11	Imputed	0.995	-	113.55 (23.37, 551.59)	**4.41×10^{-09}**	0.89	5’ of *ME3*
rs10734945	12	128295046	C/T	0.13	Imputed	0.992	-	15.92 (6.17, 41.11)	**1.07×10^{-08}**	0.79	5’ of *FLJ37505*
rs755836	12	128297785	T/C	0.13	Genotyp ed	-	-	16.02 (6.21, 41.38)	**9.97×10^{-09}**	0.79	5’ of *FLJ37505*
rs1882266	12	128298247	C/T	0.13	Imputed	0.998	-	16.02 (6.21, 41.38)	**9.97×10^{-09}**	0.79	5’ of *FLJ37505*
rs1653376	12	128301795	C/G	0.13	Imputed	0.994	-	16.07 (6.22, 41.52)	**9.84×10^{-09}**	0.81	5’ of *FLJ37505*
rs1653375	12	128301798	T/C	0.13	Imputed	0.994	-	16.07 (6.22, 41.52)	**9.84×10^{-09}**	0.81	5’ of *FLJ37505*
rs11348435	12	128303354	GA/G	0.12	Imputed	0.955	-	33.27 (9.67, 114.49)	**2.73×10^{-08}**	0.88	5’ of *FLJ37505*
rs7206003	16	75205764	A/G	0.10	Genotyp ed	-	-	95.42 (20.14, 452.01)	**9.26×10^{-09}**	0.87	3’ UTR of *ZFP1*
rs12922107	16	75206392	G/C	0.10	Imputed	0.996	-	94.6 (19.97, 448.12)	**9.88×10^{-09}**	0.87	3’ of *ZFP1*
rs12923789	16	75206439	A/G	0.10	Imputed	0.996	-	94.6 (19.97, 448.12)	**9.88×10^{-09}**	0.87	3’ of *ZFP1*
rs7188765	16	75208536	G/A	0.10	Imputed	0.962	-	93.19 (19.68, 441.37)	**1.10×10^{-08}**	0.87	3’ of *ZFP1*

rs3064467	17	63385829	TAAAC/T	0.11	Imputed	0.950	-	70.27 (17.35, 284.66)	**2.56×10^{-09}**	0.88	3’ of *AXIN2*
rs6507174	18	34121268	A/G	0.15	Imputed	0.995	-	9.34 (4.51, 19.35)	**1.85×10^{-09}**	0.45	Intron of *FHOD3*
rs1874381	18	34122410	A/G	0.15	Genotyped	-	-	9.33 (4.50, 19.33)	**1.88×10^{-09}**	0.46	Intron of *FHOD3*
rs817090	2	49575028	T/C	0.12	Imputed	0.985	Before 5 years	0.50 (0.07, 3.57)	4.87×10^{-01}	0.68	5' of *FSHR*
							After 5 years	40.45 (11.57, 141.49)	**6.96×10^{-09}**	0.80	
rs200143895	12	119707968	A/AAAAG	0.12	Imputed	0.986	Before 5 years	3.87 (0.92, 16.24)	6.42×10^{-02}	0.46	3’ of *LINC00934*
							After 5 years	141.03 (27.74, 716.86)	**2.44×10^{-09}**	0.98	
rs11064732	12	119709787	T/A	0.12	Imputed	0.995	Before 5 years	3.89 (0.93, 16.29)	6.33×10^{-02}	0.48	3’ of *LINC00934*
							After 5 years	135.71 (26.87, 685.51)	**2.81×10^{-09}**	0.97	

Chr, chromosome; CI, confidence interval; HR, hazard ratio; MAF, minor allele frequency; PH, proportional hazards. #, Hazard ratio was estimated under the recessive genetic model for AA vs [AB+BB], where A is the minor allele and B is the major allele. *, Gene annotation is derived from the UCSC database (“UCSC genes” from the UCSC browser [GRCh37/hg19]) [96], and only the overlapped (for SNPs within genes) or the closest (for SNPs in intergenic regions) genes are shown in this Table. 3’, downstream of the gene. 5’, upstream of the gene. Models are adjusted for disease stage, tumor location (3 years as the cut-off time point), adjuvant chemotherapy and radiotherapy statuses. SNPs that are in high-LD ($r^2 > 0.8$) with each other on the same chromosome are highlighted.

Supplementary Table 7. Top SNPs in multivariate analysis that have nominal/suggestive associations with recurrence/metastasis-free survival (RMFS) under the dominant and additive genetic models.

Genetic model	Variant	Chr	Position	Minor/ major allele	MAF	Variant type	Info score	Time period post-diagnosis	#HR (95% CI)	p value	p value of the PH assump-tion test
**Dominant**											
	rs1372330	9	119519588	A/G	0.14	Genotyped	-	-	2.11 (1.45, 3.05)	8.06×10^{-05}	0.22
	rs979746	17	46336112	A/C	0.19	Imputed	0.994	-	2.32 (1.61, 3.35)	7.30×10^{-06}	0.50
	rs73151111	21	25723030	G/A	0.14	Imputed	0.969	-	2.27 (1.57, 3.30)	1.43×10^{-05}	0.07
**Additive**											
	*rs71011025	2	185087530	AT/A	0.18	Imputed	0.989	-	2.22 (1.66, 2.98)	1.01×10^{-07}	0.20
	*rs13400857	2	185203547	G/T	0.19	Imputed	0.979	-	2.18 (1.63, 2.92)	1.84×10^{-07}	0.15
	*rs34039920	2	185227401	T/TAA	0.19	Imputed	0.997	-	2.22 (1.64, 2.99)	1.82×10^{-07}	0.20
	rs10160322	11	107683902	A/G	0.11	Imputed	0.977	Before 5 years	2.45 (1.70, 3.54)	1.45×10^{-06}	0.10
								After 5 years	1.17 (0.36, 3.86)	7.93×10^{-01}	0.97
	rs10160657	11	107683926	T/A	0.11	Imputed	0.985	Before 5 years	2.45 (1.71, 3.51)	1.03×10^{-06}	0.08
								After 5 years	1.08 (0.33, 3.59)	8.98×10^{-01}	0.96
	rs12808659	11	107685038	G/A	0.11	Imputed	0.989	Before 5 years	2.41 (1.68, 3.47)	1.83×10^{-06}	0.07
								After 5 years	1.02 (0.31, 3.41)	9.72×10^{-01}	0.96

Chr, chromosome; CI, confidence interval; HR, hazard ratio; MAF, minor allele frequency; PH, proportional hazards; RMFS, recurrence/metastasis-free survival. #, Hazard ratio was estimated under the dominant genetic model for [AA+AB] vs BB and under the additive genetic model for AA vs AB vs BB, where A is the minor allele and B is the major allele. *, Note that rs13400857 is in high-LD ($r^2 > 0.8$) with the other two SNPs (rs71011025 and rs34039920), but rs71011025 and rs34039920 are not in high-LD with each other ($r^2 = 0.77$). Models are adjusted for disease stage, tumor location (3 years as the cut-off time point), adjuvant chemotherapy and radiotherapy statuses. For the additive genetic model, results shown include the top three

SNPs both with and without the cut-off time point of 5 years. SNPs that are in high-LD ($r^2 > 0.8$) with each other on the same chromosome are highlighted.

Supplementary Table 8. eQTLs (identified and high-LD variants) in DSS and RMFS recessive models.

Outcome - genetic model	rs ID	*eQTL associated gene (tissue) - RegulomeDB	*eQTL associated gene (tissue) - GTEx	High-LD SNP	SNP(s) identified in our study
DSS-recessive	#rs12757197 (kgp2690683)	*TBCE* (monocyte)	*TBCE* (transverse colon)	No	rs12757197 (kgp2690683)
DSS-recessive	rs28552674	-	*TBCE* (transverse colon)	No	rs28552674
DSS-recessive	rs12758637	-	*TBCE* (transverse colon)	No	rs12758637
DSS-recessive	rs11579933	-	*TBCE* (transverse colon)	No	rs11579933
DSS-recessive	rs71640701	-	*TBCE* (transverse colon)	No	rs71640701
DSS-recessive	rs6429094	-	*TBCE* (transverse colon)	No	rs6429094
DSS-recessive	rs35242859	-	*TBCE* (transverse colon)	No	rs35242859
DSS-recessive	rs7412979	-	*TBCE* (transverse colon)	Yes	rs11579933, kgp2690683, rs12758637, rs28552674, rs35242859, rs6429094, and rs71640701

DSS-recessive	rs12726892	-	*TBCE*<br>(transverse colon)	Yes	rs11579933, kgp2690683, rs12758637, rs28552674, rs35242859, rs6429094, and rs71640701
DSS-recessive	rs7537	-	*TBCE*<br>(transverse colon)	Yes	rs11579933, kgp2690683, rs12758637, rs28552674, rs35242859, rs6429094, and rs71640701
DSS-recessive	rs34729832	-	*TBCE*<br>(transverse colon)	Yes	rs11579933, kgp2690683, rs12758637, rs28552674, rs35242859, rs6429094, and rs71640701
DSS-recessive	rs6702967	-	*TBCE*<br>(transverse colon)	Yes	rs11579933, kgp2690683, rs12758637, rs28552674, rs35242859, rs6429094, and rs71640701
DSS-recessive	rs36073314	-	*TBCE*<br>(transverse colon)	Yes	rs11579933, kgp2690683, rs12758637, rs28552674, rs35242859, rs6429094, and rs71640701
DSS-recessive	rs12087848	-	*TBCE*<br>(transverse colon)	Yes	rs11579933, kgp2690683, rs12758637, rs28552674, rs35242859, rs6429094, and rs71640701
DSS-recessive	rs6696235	-	*TBCE*<br>(transverse colon)	Yes	rs11579933, kgp2690683, rs12758637, rs28552674, rs35242859, rs6429094, and rs71640701
RMFS-recessive	rs7188765	-	*ZFP1*<br>(transverse colon)	No	rs7188765
RMFS-recessive	rs12716782	-	*ZFP1*<br>(transverse colon)	Yes	rs7206003, rs12922107, rs12923789, and rs7188765

RMFS-recessive	rs7189541	-	*ZFP1*<br>(transverse colon)	Yes	rs7206003, rs12922107, rs12923789, and rs7188765
RMFS-recessive	rs11648915	-	*ZFP1*<br>(transverse colon)	Yes	rs7206003, rs12922107, rs12923789, and rs7188765
RMFS-recessive	rs6564214	-	*ZFP1*<br>(transverse colon)	Yes	rs7206003, rs12922107, rs12923789, and rs7188765
RMFS-recessive	rs9931007	-	*ZFP1*<br>(transverse colon)	Yes	rs7206003, rs12922107, rs12923789, and rs7188765

DSS, disease-specific survival; eQTL, expression quantitative trait locus; LD, linkage disequilibrium; RMFS, recurrence/metastasis-free survival; SNP, single nucleotide polymorphism. *****, all SNPs identified in recessive models as well as those SNPs that are in high-LD with them (retrieved from Haploreg [84]) were explored in RegulomeDB [87] and GTEx [88]. Note that GTEx data are shown for colon tissue, as it has no data for rectal tissue. [#], the rs number of the identified SNP kgp2690683 is rs12757197; rs number was identified by SNP's genomic positions and alleles. The eQTLs are all cis-eQTLs that locate within ±1 Mb of the transcription start sites of the genes shown in the Table.

Supplementary Table 9. Association between *WBP11* expression levels and consensus molecular subtypes (CMS).

	P value of Kruskal-Wallis test	P value of Dunn's test for pair-wise comparison		
		Pairs	P value	Adjusted p value (Bonferroni method)
CMS (1, 2, 3, 4)	**9.66×10^{-07}**	CMS2 vs CMS1	**2.96×10^{-03}**	**1.77×10^{-02}**
		CMS3 vs CMS1	7.51×10^{-02}	4.51×10^{-01}
		CMS4 vs CMS1	**7.30×10^{-08}**	**4.38×10^{-07}**
		CMS3 vs CMS2	6.32×10^{-01}	1.00
		CMS4 vs CMS2	**1.22×10^{-03}**	**7.35×10^{-03}**
		CMS4 vs CMS3	**9.02×10^{-03}**	5.41×10^{-02}

CMS, Consensus Molecular Subtypes.

Supplementary Table 10. Top CNVs/INDELs in univariate analysis of the disease-specific survival (DSS) and recurrence/metastasis-free survival (RMFS).

Outcome	Variant	0 copy frequency	Variant type	Time period post-diagnosis	#HR (95% CI)	p value	p value of the PH assumption test
DSS							
	Chr2:227165698-227170955 (0 copy vs 1 or 2 copies)	0.11	CNV	-	2.28 (1.40, 3.73)	9.91×10^{-4}	0.73
	Chr7:73829165-73831200 (0 copy vs 2 copies)	0.30	CNV	-	1.88 (1.26, 2.80)	1.95×10^{-3}	0.10
	Chr19:15046722-15047605 (0 copy vs 2 copies)	0.28	INDEL	-	0.45 (0.26, 0.76)	3.13×10^{-3}	0.30
	Chr2:54565729-54567441 (0 copy vs 2 copies)	0.32	CNV	Before 5 years	0.68 (0.38, 1.20)	1.84×10^{-01}	1.00
				After 5 years	2.37 (1.24, 4.52)	9.02×10^{-03}	0.58
	Chr2:76773793-76775393 (0 copy vs 2 copies)	0.55	CNV	Before 5 years	0.86 (0.52, 1.42)	5.64×10^{-01}	1.00
				After 5 years	3.56 (1.56, 8.10)	2.51×10^{-03}	0.54
	Chr9:22496202-22502596 (0 copy vs 1 or 2 copies)	0.15	CNV	Before 5 years	0.73 (0.33, 1.60)	4.30×10^{-01}	0.73
				After 5 years	2.693 (1.33, 5.45)	5.94×10^{-03}	0.69
RMFS							
	Chr1:62082921-62083563 (0 copy vs 2 copies)	0.13	INDEL	-	1.97 (1.25, 3.10)	3.47×10^{-03}	0.48

	Chr2:146866008-146876881 (0 copy vs 1 or 2 copies)	0.21	CNV	-	0.48 (0.28, 0.813)	6.30×10^{-03}	0.69
	Chr7:24038309-24039976 (0 copy vs 2 copies)	0.42	CNV	-	0.58 (0.39, 0.85)	5.37×10^{-03}	0.90
	Chr2:76773793-76775393 (0 copy vs 2 copies)	0.55	CNV	Before 5 years	1.04 (0.71, 1.53)	8.34×10^{-01}	0.15
				After 5 years	2.91 (0.95, 8.92)	6.19×10^{-02}	0.89
	Chr4:172374626-172378977 (0 copy vs 2 copies)	0.11	CNV	Before 5 years	0.53 (0.25, 1.14)	1.05×10^{-01}	0.06
				After 5 years	0.94 (0.21, 4.10)	9.32×10^{-01}	0.93
	Chr22:35645524-35646052 (0 copy vs 2 copies)	0.43	INDEL	Before 5 years	0.61 (0.40, 0.91)	1.61×10^{-02}	0.19
				After 5 years	2.18 (0.81, 5.90)	1.24×10^{-01}	0.93

CI, confidence interval; CNV, copy number variation; DSS, disease-specific survival; HR, hazard ratio; INDEL, insertion/deletion; PH, proportional hazards; RMFS, recurrence/metastasis-free survival. [#], Hazard ratio was estimated for 0 copy vs at least one copy. For DSS and RMFS analyses, results shown include the top three CNVs/INDELs both with and without the cut-off time point of 5 years.
